# Supplementary material for: Data–driven modelling makes quantitative predictions regarding bacteria surface motility
Source: PLoS Comput Biol. 2024 May 14;20(5):e1012063. doi: 10.1371/journal.pcbi.1012063 (PMC11125545; doi:10.1371/journal.pcbi.1012063)
Supplement: S1 Appendix — The interactions between the bacteria and the environment surface in our model. (PDF) [file pcbi.1012063.s001.pdf]

Supporting Information

Data–driven modelling makes quantitative predictions regarding bacteria surface motility

Daniel Barton, Yow-Ren Chang, William Ducker, Jure Dobnikar

April 24, 2024

S1 Appendix. Surface Interaction

The surface of the substrate is represented by a constraint in the energy relaxation step. This constraint is implemented by a repulsive potential between the surface and the bacteria. We use a repulsive truncated Lennard Jones (WCA) potential [1].

The repulsive potential is,

$$U_R^{\mathrm{WCA}}(r) = \begin{cases} \epsilon \left[\left(\frac{R}{r}\right)^{12} - 2\left(\frac{R}{r}\right)^{6} \right] + \epsilon & r \leq R \\ 0, & r > R \end{cases} . \tag{1}$$

So that the anchor points of our TFP do not intersect the surface, we move them back inside the body by a distance $\delta_a = 0.04$ µm. For the stability of the simulation it is necessary to choose ϵ large enough that the intersection distance of the body with the surface is always less than δ_a.

For a planar surface it is sufficient to apply this interaction potential once each for the leading and trailing poles of the body. We soon find that this model exclusively generates walking behaviour due to the unbalanced torque generated by TFP activity at one pole. We therefore construct a new potential which has a short range attractive part using the Lennard Jones potential and a smoothing function,

$$S_{\mathrm{a,b}}(r) = \begin{cases} 1 & r < a \\ 1 + \left(\frac{r-a}{b-a}\right)^2 \left(2\frac{r-a}{b-a} - 3\right) & a \leq r \leq b \\ 0 & r > b \end{cases} , \tag{2}$$

so that our attractive potential is

$$U_{\text{attractive}} = U_{LJ}(r) S_{R-\delta_+,R}(r) \tag{3}$$

$$= \epsilon \left[\left(\frac{R-\delta_+}{r} \right)^{12} - 2 \left(\frac{R-\delta_+}{r} \right)^{6} \right] S_{R-\delta_+,R}(r)\,. \tag{4}$$

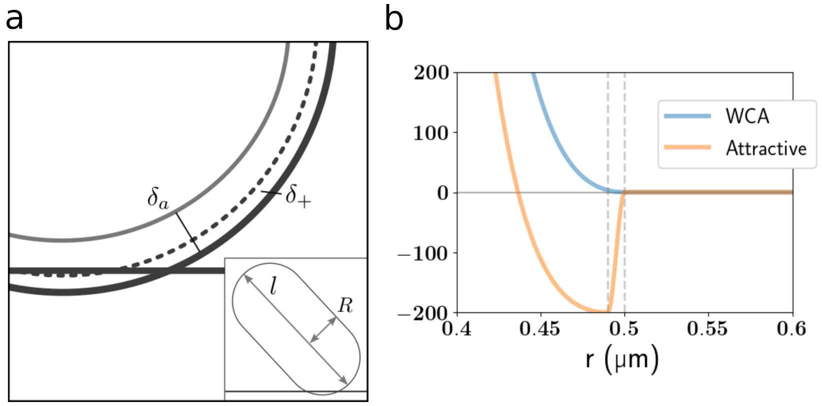

Figure 1: (a) Scaled up view of the cell surface showing the distance δ_+ used when constructing a short range surface attraction potential and the distance δ_a used to safely construct TFP so that their origin points don't intersect the surface. Inset shows the cell length l and radius R. (b) Truncated Lennard-Jones (WCA) and the attractive surface potential. Dashed vertical lines denote the region $[R-\delta_+, R]$. The ϵ parameter sets the interaction strength which in this case is $\epsilon = 200$.

The smoothing function has the effect of squashing the attractive part of the Lennard Jones potential into the tiny distance $\delta_+ = 0.01$ µm and to truncate the potential to a finite range $0 < r \leq r_{min} + \delta_+$. For the sake of simulation stability the potentials we use are designed to be continuous with continuous first derivatives. The parameter ϵ controls the depth of the potential well and the steepness of the repulsive part of the potential. Empirically, the value $\epsilon = 200$ is found to be appropriate to ensure the stability of the simulations in the repulsive (Eq.1) case. We thus use the WCA potential (Eq. 1) with $\epsilon = 200$ for simulations of the walking trajectories.

To simulate crawling behaviour, we initialise the cell with both poles in contact with the surface and use the attractive surface interaction (Eq. 3) with $\epsilon = 2 \times 10^4$. The deep attractive well in this case ensures that the surface forces are much larger than the TFP forces and that the cell body remains parallel to the surface with its center line in the plane $z = R - \delta_+$ throughout the simulation. By selecting these values, we study both motility types independently – without the need to develop a comprehensive understanding of bacteria-surface interactions. Finally, in order to study the walking-crawling transitions, we allow the surface

interaction strength to be comparable to TFP forces. In that case a typical parameter value is $\epsilon = 50$.

References

[1] John D Weeks, David Chandler, and Hans C Andersen. "Role of repulsive forces in determining the equilibrium structure of simple liquids". In: *The Journal of chemical physics* 54.12 (1971), pp. 5237–5247.
