# Supplementary material for: Data–driven modelling makes quantitative predictions regarding bacteria surface motility
Source: PLoS Comput Biol. 2024 May 14;20(5):e1012063. doi: 10.1371/journal.pcbi.1012063 (PMC11125545; doi:10.1371/journal.pcbi.1012063)
Supplement: S2 Appendix — Details of the calculations for optimising the position and orientation of a rigid body. (PDF) [file pcbi.1012063.s002.pdf]

Supporting Information

Data–driven modelling makes quantitative predictions regarding bacteria surface motility

Daniel Barton, Yow-Ren Chang, William Ducker, Jure Dobnikar

April 24, 2024

S2 Appendix. Axis–angle representation

The state of the cell body is described by an axis-angle representation [1] with the center of mass position of the body is $\boldsymbol{r}_c$ and its rotation is represented by a vector $\boldsymbol{p} = p\hat{\boldsymbol{p}}$ such that a rotation of angle $\theta = ||\boldsymbol{p}||$ around the axis $\hat{\boldsymbol{p}}$ from some reference axis $\hat{\boldsymbol{e}}^0$ yields the orientation of the body. The corresponding rotation matrix is

$$\boldsymbol{R} = \boldsymbol{I} + (1 - \cos\theta)\tilde{p}\tilde{p} + \sin\theta\,\tilde{p}$$

where $\tilde{p}$ is the skew-symmetric matrix obtained from $\boldsymbol{p}$,

$$\tilde{p} = \frac{1}{\theta}\begin{pmatrix} 0 & -p_3 & p_2 \\ p_3 & 0 & -p_1 \\ -p_2 & p_1 & 0 \end{pmatrix} .$$

The TFP anchor position in the lab frame is $\boldsymbol{r}_c + \boldsymbol{R}\boldsymbol{d}_a^0$, where $\boldsymbol{d}_a^0$ is the position of the anchor in the body frame. The body frame is defined such that the long axis of the cell coincides with $\hat{\boldsymbol{e}}^0$ and its center of mass is at the origin. The derivatives of the TFP energy are

$$\begin{aligned}
\frac{\partial u^{\mathrm{TFP}}}{\partial \boldsymbol{r}_c} &= \frac{\partial u^{\mathrm{TFP}}}{\partial r_{ab}}\frac{\partial r_{ab}}{\partial \boldsymbol{r}_c} = -\frac{E}{l_{\mathrm{eq}}}(r_{ab} - l_{\mathrm{eq}})\hat{\boldsymbol{r}}_{ab} \\
\frac{\partial u^{\mathrm{TFP}}}{\partial p_k} &= \frac{E}{l_{\mathrm{eq}}}(r_{ab} - l_{\mathrm{eq}}) \cdot \frac{\partial \boldsymbol{d}_a}{\partial p_k} \cdot \frac{(\boldsymbol{r}_b - \boldsymbol{r}_c - \boldsymbol{d}_a)}{r_{ab}} \\
&= -\frac{E}{l_{\mathrm{eq}}}(r_{ab} - l_{\mathrm{eq}})\frac{\partial \boldsymbol{R}}{\partial p_k}\boldsymbol{d}_a^0 \cdot \hat{\boldsymbol{r}}_{ab}\,,
\end{aligned}$$

where $\hat{\boldsymbol{r}}_{ab}$ is the normalized vector $(\boldsymbol{r}_a - \boldsymbol{r}_b)/r_{ab}$ and p_k is the kth component of $\boldsymbol{p}$. Since pili cannot push the cell by extension, this calculation is for $r_{ab} < l_{\mathrm{eq}}$, otherwise the energy associated with the pilus is 0. To solve for the new position of the body we minimise the energy with the respect to the 6-vector $(\boldsymbol{r}_c, \boldsymbol{p})$. The

fast inertial relaxation (FIRE) [2] algorithm was implemented to perform this optimisation.

References

[1] Dwaipayan Chakrabarti and David J Wales. “Simulations of rigid bodies in an angle-axis framework”. In: *Physical Chemistry Chemical Physics* 11.12 (2009), pp. 1970–1976.

[2] Erik Bitzek et al. “Structural relaxation made simple”. In: *Physical review letters* 97.17 (2006), p. 170201.
