# Supplementary material for: Data–driven modelling makes quantitative predictions regarding bacteria surface motility
Source: PLoS Comput Biol. 2024 May 14;20(5):e1012063. doi: 10.1371/journal.pcbi.1012063 (PMC11125545; doi:10.1371/journal.pcbi.1012063)
Supplement: S3 Appendix — Details of the calculations for a sampling TFP configurations with respect to their Boltzmann weight. (PDF) [file pcbi.1012063.s003.pdf]

Supporting Information

Data–driven modelling makes quantitative predictions regarding bacteria surface motility

Daniel Barton, Yow-Ren Chang, William Ducker, Jure Dobnikar

April 24, 2024

S3 Appendix. TFP Generator

Unbound TFP are modelled as discrete worm-like chains with persistence length $L_p = 5$ µm [1]. Instead of simulating the dynamics of flexible TFP, we approximately model their flexibility by regularly updating the configuration of pili using a Boltzmann generator with a resampling rate k_{resample}. A Boltzmann generator is a procedure that generates states of a system with a frequency that is proportional to their Boltzmann weight.

Worm-like chains are composed of n segments of length a with Hamiltonian

$$\mathcal{H} = -\frac{k_a}{a} \sum_{i=1}^{n} \cos \phi_i \, ,$$

where $\{\phi_i\}$ are the polar coordinates, $\{\theta_i\}$ are the azimuthal coordinates and k_a is the bending stiffness. k_a is related to the persistence length L_p by $k_a = k_B T L_p$, where k_B is the Boltzmann constant and T is the temperature. We can change coordinates using $\phi_i = \cos^{-1}(aE_i/k_a)$, where E_i is the energy of segment i.

The probability that the system is in a microstate with energy E is $P(E) = g(E)\exp(-\beta E)/\mathcal{Z}$ where $g(E)$ is the density of states and $\mathcal{Z}$ is the partition function. In this simplified model, the n segments of the chain are mutually independent which means we can generate a chain by sampling the ϕ distribution for a single segment n times. The microstates of a single chain segment are arranged on the surface of a sphere with radius a and surface element $dS_a = a^2 \sin\phi_i d\phi_i d\theta_i$. Using $\phi_i = \cos^{-1}(aE_i/k_a)$, which is a one to one mapping on $\phi \in [0, \pi]$,

$$g(E_i) = 2\pi a^2 \sin\phi_i \, d\phi_i = 2\pi a^3/k_a \, dE_i \, ,$$

which doesn't depend explicitly on E. Hence, the energy of a segment is simply proportional to $\exp(-\beta E)$, which we can sample numerically. In this work we use $a = 0.2$ µm and restrict ϕ angles to the range $[0, \pi/2]$. For these values of

a and L_p, the probability of $\phi > \pi/2$ is vanishingly small. Inverse transform sampling is used to generate the ϕ_i coordinates and we draw the θ_i coordinates from a uniform distribution.

References

[1] Jeffrey M Skerker and Howard C Berg. "Direct observation of extension and retraction of type IV pili". In: *Proceedings of the National Academy of Sciences* 98.12 (2001), pp. 6901–6904.
