# Supplementary material for: Data–driven modelling makes quantitative predictions regarding bacteria surface motility
Source: PLoS Comput Biol. 2024 May 14;20(5):e1012063. doi: 10.1371/journal.pcbi.1012063 (PMC11125545; doi:10.1371/journal.pcbi.1012063)
Supplement: S4 Appendix — (PDF) [file pcbi.1012063.s004.pdf]

Supporting Information

Data–driven modelling makes quantitative predictions regarding bacteria surface motility

Daniel Barton, Yow-Ren Chang, William Ducker, Jure Dobnikar

April 24, 2024

S4 Appendix. Angular flexibility vs. retraction speed

The assumption underlying the choice of v_{ret} as a free parameter in our model is based on a few basic assumptions about the motility mechanism. First, we assume that the dissipation forces are smaller than the those that the motors are capable to exert. Second, the motor works so that it adjusts the exerted force, which equals the tension in TFP such that the tension is balanced with the dissipative forces. Given that the retraction speed has been observed to depend on the tension, as a result, the TFP retraction speed is defined by the magnitude of the friction and viscous forces. We don't know the magnitudes of these forces to any kind of acceptable precision, therefore we rather assume v_{ret} to be a free parameter and optimize its value through Bayesian computation (see main text). To do this, we additionally assumed that the surface is homogeneous and consequently v_{ret} is approximately constant for a given trajectory.

Following the procedure based on these assumptions, we determined that the retraction speed of TFP at the experimental conditions of the analyzed tracking data [1] is around 0.17 μms^{-1}, much lower than the typical previously assumed value in the range $0.5 - 1$ μms^{-1}, but in good agreement with recent experimental observations [2] measuring 0.09 μms^{-1} at conditions very similar to those at which the tracking data was obtained. Even so, we investigated another possibility, that the TFP retraction speed is inhibited at large angles relative to the bacteria surface. For the sake of completeness we include it here.

Bound TFP have their anchor positions fixed relative to the cell envelope and attachment positions fixed on the surface. It follows that as a TFP retracts, the angles it makes with the cell body and with the surface varies. This effect may become extreme for large angle between the pilus and the cell body surface normal, θ_a. To avoid this, we introduce a model parameter $\alpha \in [0, \pi/2]$ that prevents the TFP machine driving retraction at large angles. The modified retraction velocity is $v'_{\text{ret}}(\theta_a) = v_{\text{ret}} S_\alpha(\theta_a)$, where the function $S_\alpha(\theta) = 1 + \left(\frac{\theta}{\alpha}\right)^2 \left(2\frac{\theta}{\alpha} - 3\right)$ decays smoothly to zero on $[0, \alpha]$, for $\theta > \alpha$ we set $S_\alpha(\theta) = 0$. TFP may still attain $\theta_a > \alpha$ at the moment of binding the surface and by the

movement of the body due to other TFP but the pilus will not be driven into this state by its own a retraction motor.

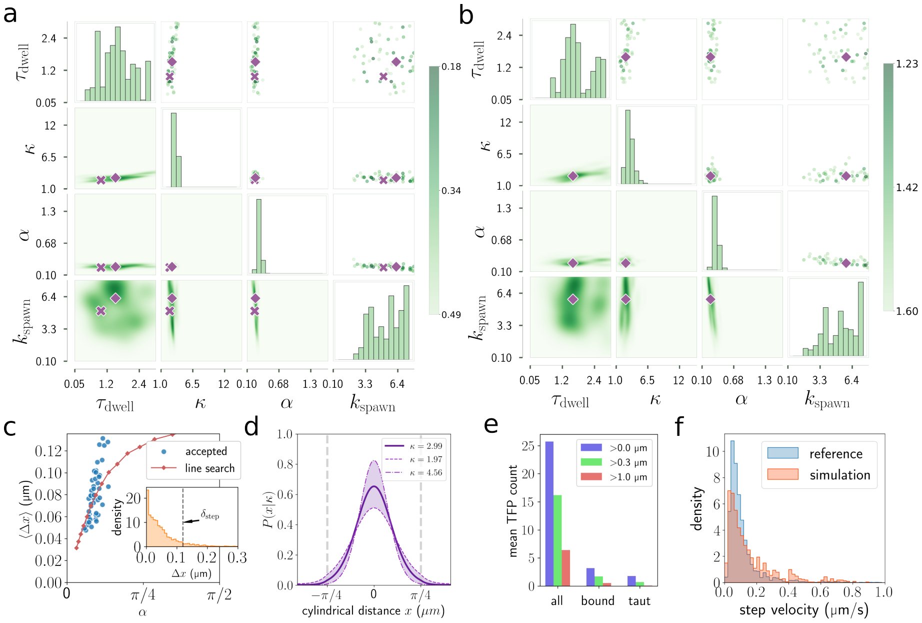

Figure 1: **Parameter inference for crawling trajectories.** (a,b) Cardinal projections of the 4d approximate posterior distribution obtained by accepting 50/10000 samples. The reference data for (a) is simulated data and for (b) it is a subset of the tracking data. Weighted histograms of the accepted parameter values are plotted on the diagonal axes. Crosses mark the parameters of the simulated reference data while Diamonds show the maximum likelihood estimates. Color indicates the sample score, ρ. (c) Blue circles are the mean per-TFP displacements, $\langle \Delta x \rangle$, of the accepted samples. The inset figure is the Δx distribution for the best scoring simulation. (d) Probability density function used for generating TFP on the body surface. The distribution is extended onto the cylindrical portion of the spherocylinder. Shaded region is the 90% confidence interval in the ABC estimate of the κ parameter. (e) Counting TFP using the best scoring simulation. the mean numbers of total TFP, bound TFP and taut TFP are shown as well as those counts in the cases that only TFP longer than 0.3 µm and 1.0 µm are visible to the observer. (f) Linearised velocity distributions for experimental tracking data (blue) and the best scoring simulated data (orange).

We ran the same Bayesian computation as in the main text for the model with fixed retraction speed of 0.5 µms^{-1}, and with variable α. The results (Fig. S2) demonstrate that qualitatively all the conclusions discussed within the original approach remain the same, which suggests that in principle this could be an

alternative motility mechanism. However, we stress again that the assumption of renormalized constant retraction speed is supported by experimental work, while there is no experimental evidence supporting the model presented in this Appendix.

References

[1] Fan Jin et al. "Bacteria use type-IV pili to slingshot on surfaces". In: *Proceedings of the National Academy of Sciences* 108.31 (2011), pp. 12617–12622.

[2] Jingchao Zhang et al. "A simple, switchable pili-labelling method by plasmid-based replacement of pilin". In: *Environmental Microbiology* 23.5 (2021), pp. 2692–2703.
