# Supplementary material for: Data–driven modelling makes quantitative predictions regarding bacteria surface motility
Source: PLoS Comput Biol. 2024 May 14;20(5):e1012063. doi: 10.1371/journal.pcbi.1012063 (PMC11125545; doi:10.1371/journal.pcbi.1012063)
Supplement: S5 Appendix — Introduction to the sensitivity analysis method used in this work. (PDF) [file pcbi.1012063.s005.pdf]

Supporting Information

Data–driven modelling makes quantitative predictions regarding bacteria surface motility

Daniel Barton, Yow-Ren Chang, William Ducker, Jure Dobnikar

April 24, 2024

S5 Appendix. Sensitivity analysis

Sensitivity analysis is a collection of tools which operate on a function of many parameters $u = f(\boldsymbol{x}) = f(x_1, \ldots, x_n)$ to estimate the influence on f of each parameter x_i independently. One method that immediately comes to mind is to choose some input $\boldsymbol{x}'$ and estimate the partial derivatives $(\partial u/\partial x_i)|_{\boldsymbol{x}=\boldsymbol{x}'}$, in other words the sensitivity of $f(\boldsymbol{x})$ locally around $\boldsymbol{x}'$.

We would instead prefer to explore the behaviour of our twitching model across some broad range of input parameters. Computing the sensitivity of $f(\boldsymbol{x})$ for x_i in some bounds $[x_i^-, x_i^+]$ forming a bounded interval $\mathcal{I}$. This is the remit of global sensitivity analysis. We choose the Sobol[1] method and describe it here.

Sobol starts with a generic decomposition of $f(\boldsymbol{x})$,

$$f(\boldsymbol{x}) = f_0 + \sum_i f_i(x_i) + \sum_{i<j} f_{ij}(x_i, x_j) + ... + f_{12\ldots n}(x_1, x_2, \ldots, x_n)\,, \tag{1}$$

where the 0th term, f_0, is the integral of f on our bounded interval $\mathcal{I}$ and the kth sum is over all $\binom{n}{k}$ combinations of input variables. Let $\boldsymbol{x}_w$ be any such combination of variables and $\boldsymbol{i}_w$ be the corresponding indices. The functions $f_{\boldsymbol{i}_w}$ are variations with respect to $\boldsymbol{x}_w$ of f around f_0 in the sense that $\int f_{\boldsymbol{i}_w}(\boldsymbol{x}_w) dx_{\boldsymbol{i}_w} = 0$.

Sobol showed that the variance $V = \int_{\mathcal{I}} f^2 d\boldsymbol{x} - f_0^2$ can be similarly decomposed

$$V = \sum_i V_i + \sum_{i<j} V_{ij} + ... + V_{12\ldots n}\,, \tag{2}$$

where $V_{\boldsymbol{i}_w} = \int f_{\boldsymbol{i}_w}^2 d\boldsymbol{x}_w$ is the part of the variance of f associated with $f_{\boldsymbol{i}_w}$.

The Sobol global sensitivity indices are the normalized variances $S_{\boldsymbol{i}_w} = V_{\boldsymbol{i}_w}/V$. Furthermore, for any subset of variables $\boldsymbol{y} = (x_{k_1}, \ldots, x_{k_m}), m < n$, with complimentary subset $\boldsymbol{z}$ such that $\boldsymbol{x} = (\boldsymbol{y}, \boldsymbol{z})$, let $V_{\boldsymbol{y}}$ be the sum of all terms in eq.2

whose indices are all contained in the set $\{k_1, \ldots, k_m\}$ and $\widetilde{V}_{\boldsymbol{y}}$ be the complementary sum so that $V = V_{\boldsymbol{y}} + \widetilde{V}_{\boldsymbol{y}}$. $\widetilde{V}_{\boldsymbol{y}}$ turns out to be a useful measure of the global total variance associated with the complementary subset $\boldsymbol{z}$ and vice versa. We can write the total sensitivity index of $\boldsymbol{y}$ as $S^T_{\boldsymbol{y}} = \widetilde{V}_{\boldsymbol{z}}/V = (V - V_{\boldsymbol{z}})/V$.

For our analysis in particular, we are satisfied with computing one total sensitivity index for each variable $S^T_i = S^T_{\boldsymbol{y}}|_{\boldsymbol{y}=x_i}$. This can be done by evaluating N uniform random samples $f(\boldsymbol{x}^j)$ for $\boldsymbol{x}^j \in \mathcal{I}$ and an additional N non-random samples for each of the n variables for a total of $N(n+1)$ evaluations. The additional samples are $f(\hat{x}^j_i, \boldsymbol{x}^j_{\sim i})$ where $\boldsymbol{x}_{\sim i}$ are the the original random samples, $\boldsymbol{x}^j$, not including x_i and $\hat{x}^j_i$ is a new random sample of x_i. The original random samples are used to estimate V, then the sensitivity indices are computed as

$$S^T_i = \frac{1}{V}\frac{1}{2N}\sum_j \left(f(\boldsymbol{x}^j) - f(\hat{x}^j_i, \boldsymbol{x}^j_{\sim i}) \right)^2 . \quad (3)$$

For details of the efficient estimation of $V_{\boldsymbol{y}}, \widetilde{V}_{\boldsymbol{y}}$ see Saltelli et al.[2]. As a general rule Eq. 3 converges faster using a sampling scheme that distributes points evenly in the sampling space than for uniform random sampling [1], we use a sobol sequence. We make use of the SALib[3] library where these algorithms are implemented.

References

[1] Ilya M Sobol. "Global sensitivity indices for nonlinear mathematical models and their Monte Carlo estimates". In: *Mathematics and computers in simulation* 55.1-3 (2001), pp. 271–280.

[2] Andrea Saltelli et al. "Variance based sensitivity analysis of model output. Design and estimator for the total sensitivity index". In: *Computer physics communications* 181.2 (2010), pp. 259–270.

[3] Jon Herman and Will Usher. "SALib: An open-source Python library for Sensitivity Analysis". In: *The Journal of Open Source Software* 2.9 (Jan. 2017). DOI: 10.21105/joss.00097. URL: https://doi.org/10.21105/joss.00097.
