# Supplementary material for: Data–driven modelling makes quantitative predictions regarding bacteria surface motility
Source: PLoS Comput Biol. 2024 May 14;20(5):e1012063. doi: 10.1371/journal.pcbi.1012063 (PMC11125545; doi:10.1371/journal.pcbi.1012063)
Supplement: S6 Appendix — Analysis showing that it is difficult for our methods to differentiate between different types of surface sensing behaviour that have been suggested for P. aeruginosa. (PDF) [file pcbi.1012063.s006.pdf]

Supporting Information

Data–driven modelling makes quantitative predictions regarding bacteria surface motility

Daniel Barton, Yow-Ren Chang, William Ducker, Jure Dobnikar

April 24, 2024

S6 Appendix. Surface Sensing

Using state of the art microscopy techniques, Tala et al. were able to measure the surface dwell time of of TFP for several *P. Aeruginosa* mutants [1]. We are particularly interested to see that the the TFP of the fliC$^-$ pilT$^-$ mutant, which lacks the necessary proteins to make either flagella or the PilT retraction motor, exhibit a short median dwell time of 75 ms. The fliC$^-$ mutant with the PilT motor has, in contrast, a TFP median dwell time of 1.0 s. Tala et al. conclude that this discrepancy is evidence of a mechanism whereby tension caused by TFP retraction triggers a stronger surface binding. Let the delay between surface contact and retraction be τ_{delay}, we expect an efficient system of twitching motility provided that $\tau_{\mathrm{delay}} \ll \tau_{\mathrm{dwell}}$, in other words that TFP retraction occurs shortly after surface contact. Tala et al. find the median delay time $\tau_{\mathrm{delay}} = 135$ ms, they suggest that rapid retraction may be triggered by a surface sensing mechanism and provide some evidence for it.

Another investigation, this one using an optical trap to contain bacteria while TFP extension/retraction behaviours are measured, argue that the surface sensing mechanism is not necessary to explain the retraction of TFP[2]. We make heavy use of the results of both works in our modelling. Koch et al. use an exclusive binding model of extension and retraction motors to obtain estimates for the binding and unbinding rates of these motors. Their estimates of the unbinding rate of the extension motor and subsequent binding rate of the retraction motor are $k_{\mathrm{ext,off}} = 1.6$ s and $k_{\mathrm{ret,on}} = 0.4$ s respectively. Note that the conclusions of these two investigations are not directly contradictory, since the surface sensing mechanism may exist even though it is not necessary to explain the results of Koch et al.

Our work is opinionated on the subject. We accept the Tala et al. result that $\tau_{\mathrm{delay}} \ll \tau_{\mathrm{dwell}}$, otherwise twitching motion may be inefficient. Since τ_{delay} is short compared to the other timescales in our model, we use the approximation $\tau_{\mathrm{delay}} \to 0$, which in the language of motor binding and unbinding rates implies that $1/k_{\mathrm{ext,off}} + 1/k_{\mathrm{ext,on}} \to 0$ specifically for TFP in contact with the

surface. We otherwise accept the estimates for $k_{\mathrm{ext,off}}, k_{\mathrm{ext,on}}$ from Koch et al. for unbound TFP.

We briefly investigated whether any information about surface sensing behaviour can be inferred from tracking data alone using our model. To do this, we set $1/k_{\mathrm{ext,off}} \rightarrow 0$ for surface bound TFP as before, to prevent TFP from being driven into the surface by the extension motor. We then free up $k_{\mathrm{ret,on}}$ to vary in the range $[0.4\ \mathrm{s}, 4.0\ \mathrm{s}]$ to mimic the effect of a varying τ_{delay}. Surprisingly we do not see large differences in the characteristics of our simulated trajectories by varying $k_{\mathrm{ret,on}}$ in this variant of our model. A sensitivity analysis indicates why this is the case (table 1).

Firstly see that the indices for the $k_{\mathrm{ret,on}}$ parameter for observable statistics are all small except for $\hat{q}$ which is only 0.09, supporting our cursory observation that varying the parameter did not have a large effect on the trajectories. If we want to pursue this line of inquiry further we should focus on the $\hat{q}$ statistic to distinguish surface sensing models. Secondly, the mean TFP binding number $\langle N_{\mathrm{bound}} \rangle$ changes significantly with $k_{\mathrm{ret,on}}$ but $\langle N_{\mathrm{taut}} \rangle$ does not, indicating that although the number of surface bound TFP is varying significantly, the typical number of TFP that are actively contributing to the dynamics is not. These additional passive TFP are typically not in either extension or retraction states, the cell still experiences the effect of these passive TFP but only when they become taut by the active motion of the cell.

	min	max	$\langle u \rangle$	$Var(\theta_d)$	$\hat{q}$	$\hat{a}$	$\langle N_{\mathrm{bound}} \rangle$	$\langle N_{\mathrm{taut}} \rangle$
$k_{\mathrm{ext,off}}$	0.2	1	0.2695	0.0297	0.1997	0.0303	0.2662	0.1348
τ_{dwell}	0.5	3	0.1287	0.0912	0.2249	0.0297	0.2388	0.4091
κ	1	20	0.0747	0.7794	0.4330	0.0671	0.0282	0.2383
α	0.125	1	0.3391	0.1249	0.3870	0.9514	0.0038	0.0863
k_{spawn}	0.5	5	0.3846	0.0736	0.1266	0.0279	0.3630	0.3439
$k_{\mathrm{ret,on}}$	0.25	2.5	0.0236	0.0149	0.0892	0.0201	0.3165	0.0372

Table 1: Sensitivity indices for a search interval containing $k_{\mathrm{ret,on}}$ and five other parameters. Indices are shown for 4 summary statistics that can be obtained from tracking data as well as the mean number of bound $\langle N_{\mathrm{bound}} \rangle$ and the mean number of taut $\langle N_{\mathrm{taut}} \rangle$ TFP.

References

[1] Lorenzo Talà et al. “Pseudomonas aeruginosa orchestrates twitching motility by sequential control of type IV pili movements”. In: *Nature microbiology* 4.5 (2019), pp. 774–780.

[2] Matthias D Koch et al. “Competitive binding of independent extension and retraction motors explains the quantitative dynamics of type IV pili”. In: *Proceedings of the National Academy of Sciences* 118.8 (2021).
