# Supplementary material for: Data–driven modelling makes quantitative predictions regarding bacteria surface motility
Source: PLoS Comput Biol. 2024 May 14;20(5):e1012063. doi: 10.1371/journal.pcbi.1012063 (PMC11125545; doi:10.1371/journal.pcbi.1012063)
Supplement: S7 Appendix — The maximum likelihood estimators for random walk parameters that we use are sensitive to extremely large fluctuations in velocity. The problem is solved by setting a velocity threshold in our calculations. (PDF) [file pcbi.1012063.s007.pdf]

Supporting Information

Data–driven modelling makes quantitative predictions regarding bacteria surface motility

Daniel Barton, Yow-Ren Chang, William Ducker, Jure Dobnikar

April 24, 2024

S7 Appendix. Robustness of random walk statistics

The standard approach to parameter inference by approximate Bayesian computation is to compare high dimensional data, in our case trajectory data, using summary statistics[1]. In order to obtain two such statistics which have meaningful interpretations, we use the idea of Metzner et al.[2] and compare our data against one of the simplest models for bacteria motion, a persistent random walk. The random walk is described by $\boldsymbol{v}_i = q\boldsymbol{v}_{i-1} + a\boldsymbol{n}_i$ where $\boldsymbol{n}_i$ are sampled from the two dimensional normal distribution with zero mean and unit variance. The maximum likelihood estimators for q, a are

$$\hat{q} = \sum_i \boldsymbol{v}_i \cdot \boldsymbol{v}_{i-1} / \sum_i \boldsymbol{v}_{i-1} \cdot \boldsymbol{v}_{i-1} \,, \quad \hat{a} = \sqrt{\frac{1}{2(n-1)} \sum_i \left(\boldsymbol{v}_i - \hat{q}\boldsymbol{v}_{i-1}\right)^2} \,.$$

Due to the presence of experimental noise at short length- and timescales, we coarse grain our trajectories using a reference length of $\delta_{\text{step}} = 0.12$ µm. The instantaneous velocity $\boldsymbol{v}_i$ is computed relative to this coarse graining, with the indices i corresponding to linear steps of at least δ_{step}. In the following, we analyse a data set of 63 crawling trajectories. The mean instantaneous velocities of individual trajectories in this data set after coarse graining range from 0.057 µm/s to 0.130 µm/s. The total number of coarse grained velocity measurements is $N_{\text{exp}} = 8729$.

We assess the robustness of the estimators $\hat{q}, \hat{a}$ by splitting this data into n chunks of size N and computing n independent estimates. We expect that the means of our independent estimates, call them $\bar{q}, \bar{a}$, should be nearly identical to a single estimate containing the whole data set. That is, $\bar{q} \cong \hat{q}$ where

$$\bar{q} = \frac{1}{n} \sum_{k=0}^{n} \sum_{i=kN}^{(k+1)N} \frac{\boldsymbol{v}_i \cdot \boldsymbol{v}_{i-1}}{\boldsymbol{v}_{i-1} \cdot \boldsymbol{v}_{i-1}}$$

and $\bar{a}$ is defined similarly.

The results of $\bar{q}, \bar{a}$ for varying N are shown in figure 1. Not only is $\bar{q}$ not equal to $\hat{q}$, the number varies with N. In contrast, typical simulated data generated with parameters $(\tau_{\text{dwell}}, \kappa, \alpha, k_{\text{spawn}}) = (1.0\text{ s}, 2.5, 0.24, 5.0\text{s}^{-1})$ does not have this issue. We therefore examine the $\boldsymbol{v}_i \cdot \boldsymbol{v}_{i-1}$ term to identify the problem.

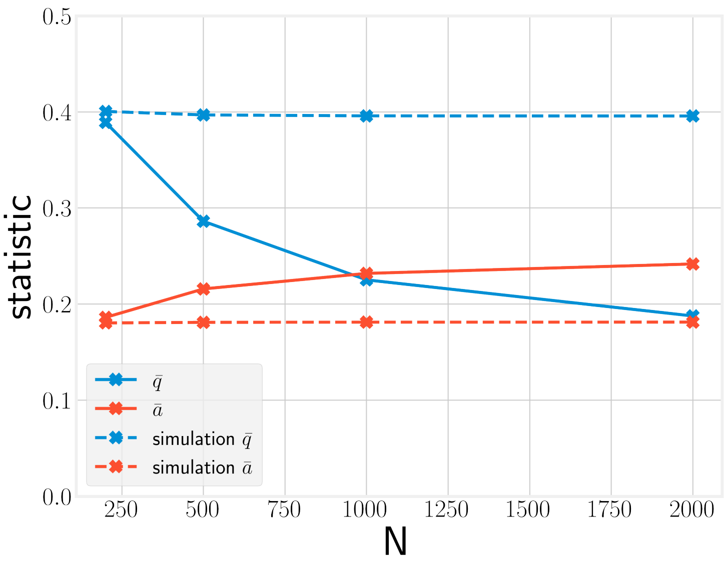

Figure 1: The mean of a set of independent estimates for the persistent random walk parameters q, a, obtained by splitting our trajectory velocity data into independent chunks of size N.

We see that the dot product of experimental velocities has large outliers (Figure 2). In fact the minimum and maximum values are $(-11.87, 14.71)$ while the limits for the simulated data were $(-0.073, 1.82)$. We believe that these outliers are the product of errors in the image analysis and tracking algorithms and do not represent the true instantaneous velocities of the bacterium. We therefore set a threshold on the instantaneous speed used in our calculation of this pair of statistics. We choose the threshold $v_i < 1.0$ µm/s, which is an order of magnitude larger than the mean instantaneous velocities of these trajectories. However because our experimental trajectories as a whole vary significantly in their mean velocity, we find it convenient to instead threshold out the top 1% of velocities. Our threshold is then the 99th percentile of the velocity distribution, which for the experimental data shown here is 0.93 µm/s. We apply this 1% threshold to simulated and experimental data, and find that we now obtain consistent estimates with respect to N of $(\bar{q}, \bar{a}) = (0.57, 0.083)$ for this experimental data and $(\bar{q}, \bar{a}) = (0.42, 0.17)$ for the simulated data shown here. We apply the threshold consistently across simulated and experimental whenever we calculate these statistics.

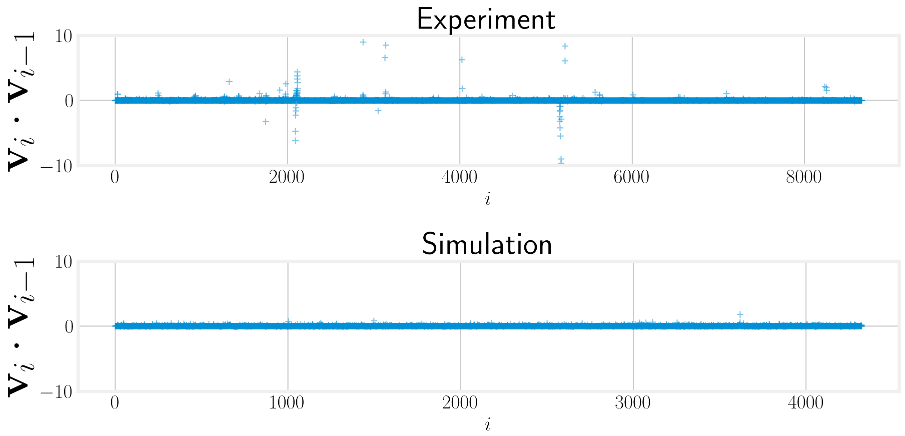

Figure 2: Large outliers, both positive and negative, can be seen in the dot product $\boldsymbol{v}_i \cdot \boldsymbol{v}_{i-1}$ for experimental data but not simulated data.

References

[1] Mark A Beaumont. "Approximate Bayesian computation in evolution and ecology". In: *Annual review of ecology, evolution, and systematics* 41 (2010), pp. 379–406.

[2] Claus Metzner et al. "Superstatistical analysis and modelling of heterogeneous random walks". In: *Nature communications* 6.1 (2015), pp. 1–8.
