# Supplementary material for: Data–driven modelling makes quantitative predictions regarding bacteria surface motility
Source: PLoS Comput Biol. 2024 May 14;20(5):e1012063. doi: 10.1371/journal.pcbi.1012063 (PMC11125545; doi:10.1371/journal.pcbi.1012063)
Supplement: S8 Appendix — The extracellular polymers may in turn modify the motility characteristics of the bacteria. The experiments we study are relatively short and we do not see significant variations in motility that could be attributed to surface deposits of EPS. (PDF) [file pcbi.1012063.s008.pdf]

Supporting Information

Data–driven modelling makes quantitative predictions regarding bacteria surface motility

Daniel Barton, Yow-Ren Chang, William Ducker, Jure Dobnikar

April 24, 2024

S8 Appendix. Surface adaptation and Psl trails

It has been observed that *P. Aeruginosa* modifies its local micro-environment by depositing the exopolysaccharide, Psl. The bacteria are then observed to preferentially follow Psl trails while traversing the surface [1]. Compared to experiments on trail following, the experimental data we study was recorded in a relatively short time frame. The bacteria were exposed to the surface and left for a few minutes to attach, and subsequently tracked for 30 minutes. We therefore expected relatively little build up of Psl or other excreted chemicals in this experiment. To examine this assumption, we reconstructed the field of view of the microscope (Figure 1a). The figure shows the trails of 785 bacteria, approximately one fifth of the total experimental data (the experimental data was gathered during five replicates of the same tracking experiment). For simplicity, we suppose that bacteria deposit Psl on the surface in a circle with radius $R = 0.5$ µm at the position of their leading pole. We then follow the time evolution of the trajectories and on each frame, increment the pixel values in a circle around the leading pole of the bacteria. This procedure gives a rough estimate of where the Psl trails would be on the surface. We then follow the time evolution of each trajectory individually and compute its overlap with all the other trails. Let A be the number of pixels in a trail and let B be the number of pixels in that trail that overlap other trails, then define the overlap fraction as B/A.

To understand whether overlapping with previous trails influences the motility characteristics, we look for correlations between the summary statistics that we use to analyse trajectory data and the overlap fraction. The statistics used are the mean velocity, the variance of the deviation angle, the persistence and the activity. We see no correlation between the overlap fraction and the first two statistics. At particularly high overlap fraction, there are slight correlations between overlap fraction and the persistence and activity statistics. Note that not all trajectories are recorded for the full 30 minute duration of the experiment. The overlap fraction of a trajectory increases with its initial time, since there

is more time for Psl trails to build up on the surface. However, this means that in general we cannot say whether variations in trajectories are due to Psl or some other process by which the bacteria might be adapting to their new surface environment. In summary, we typically do not see any strong influence of the trails made by these bacteria on each other in this experiment.

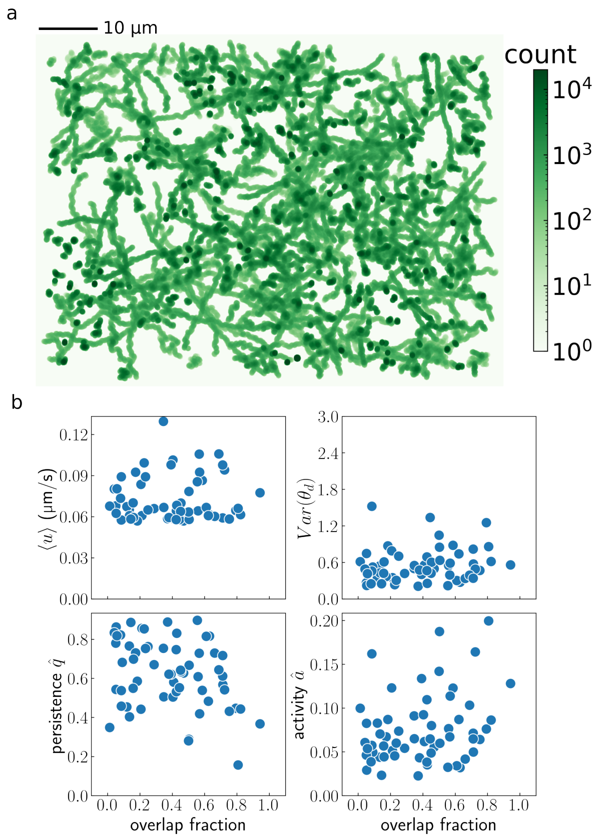

Figure 1: (a) Bacteria trails. Pixel values are the number of frames that the leading pole of the bacteria is close to that pixel. (b) Scatter plot for each summary statistic vs. overlap fraction (only for crawling trajectories, trajectories are selected using the criteria described in the manuscript.).

References

[1] Kun Zhao et al. “Psl trails guide exploration and microcolony formation in Pseudomonas aeruginosa biofilms”. In: *Nature* 497.7449 (2013), pp. 388–391.
