# Supplementary material for: Data–driven modelling makes quantitative predictions regarding bacteria surface motility
Source: PLoS Comput Biol. 2024 May 14;20(5):e1012063. doi: 10.1371/journal.pcbi.1012063 (PMC11125545; doi:10.1371/journal.pcbi.1012063)
Supplement: S9 Appendix — The aspect ratio of the surface projected bodies of the bacteria are used to identify crawling and walking behaviour. The robustness of this method is discussed and examples of aspect ratio time series are shown. (PDF) [file pcbi.1012063.s009.pdf]

Supporting Information

Data–driven modelling makes quantitative predictions regarding bacteria surface motility

Daniel Barton, Yow-Ren Chang, William Ducker, Jure Dobnikar

April 24, 2024

S9 Appendix. Aspect ratio profiles

To identify walking and crawling states and the transitions between the states, we analyse variations in the aspect ratio $b = \text{length}/\text{width}$. At the original 0.1 s resolution of the experiment, there is measurement noise in the value of the aspect ratio, so we coarse grained it using a sliding window of 20 s (200 frames), call the coarse grained time series b_t with minimum value $b_{\text{min}} = \min(b_t)$. We expect most walking trajectories to have b_{min} close to 1 because they eventually pass through a nearly vertical state during twitching. In the main text, we equate trajectories with $b_{\text{min}} < 1.6$ as walking and those with $b_{\text{min}} > 1.6$ as crawling. The $b_{\text{min}} < 1.6$ threshold selects 371 out of 3113 trajectories that exhibit some walking behaviour. We also select 100 trajectories with similar mean velocities from the $b_{\text{min}} > 1.6$ (crawling) subset and check them by eye for possible transitions between walking and crawling or other aberrations. A small handful of trajectories appear to transition between walking and crawling states, which we removed and recorded in a separate list. From the remaining trajectories, we manually filtered out 175/371 walking and 63/100 crawling trajectories and used these subsets for our analysis.

We plotted the aspect ratio (b_t) time series to evaluate whether the trajectories in the crawling and walking subsets are correctly identified. For convenience, we sort the trajectories by the variance of the aspect ratio, $\text{Var}(b_t)$, with the idea that trajectories that transition between walking and crawling will have high variance. Figure 1 shows the distributions of $\text{Var}(b_t)$ for the 175 walking trajectories and 63 crawling trajectories. Naturally, walking trajectories have a higher variance on average.

Aspect ratio profiles for trajectories in the crawling data set with median $\text{Var}(b_t)$ and max $\text{Var}(b_t)$ are shown in Figure 2. Like most crawling trajectories, the median trajectory has a fairly constant aspect ratio while the other shows some indications that it makes out-of-plane rotations for a small part of its duration. Apart from the first ∼50 s the max $\text{Var}(b_t)$ trajectory presents like a crawling trajectory, with high directional persistence and fairly constant aspect

ratio. Although we could further purify the collection of crawling trajectories by removing a few trajectories with high $\mathrm{Var}(b_t)$, doing so does not significantly affect the average statistics. We therefore avoid adding complications to our data preprocessing procedures that do not make a significant impact on the results.

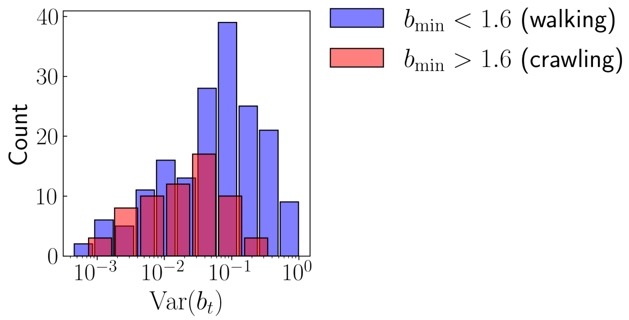

Figure 1: Distributions of the variance of the aspect ratio b = length/width, which is coarse grained using a sliding window of 20 s (200 frames), call this time series b_t. In the paper, we separate trajectories using a threshold of $b_{\min} = 1.6$ where $b_{\min}$ is the minimum of the coarse grained aspect ratio.

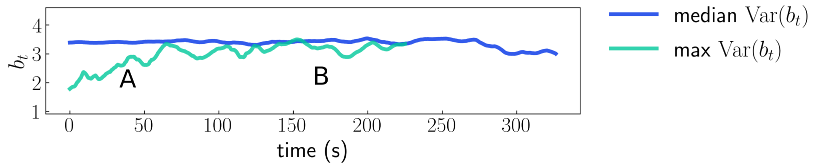

Figure 2: Coarse grained aspect ratio time series for the median $\mathrm{Var}(b_t)$ trajectory and the the maximum $\mathrm{Var}(b_t)$ trajectory. The latter trajectory may be in the walking state for the first $\sim$50 s (marker A) and then transition to the crawling state (marker B). The variations in b_t in the vicinity of marker B might be due to small out-of-plane movements, or they could be simply be due to the tracking algorithm. Removing a few trajectories with high $\mathrm{Var}(b_t)$ from the crawling subset does not significantly affect our results.

Figure 3 shows examples of the aspect ratio time series for walking trajectories, sorted by $\mathrm{Var}(b_t)$. The top half of the figure shows the four time series with the largest $\mathrm{Var}(b_t)$ (where necessary, time series are truncated to 300 s so that they fit in the figure.) The bottom half shows more typical walking trajectories. We are not concerned that a very small number of trajectories with high $\mathrm{Var}(b_t)$ show evidence of partial crawling behaviour (regions highlighted in blue) because we checked that removing a few such trajectories from the subset does not significantly affect our results. Interestingly, a few trajectories with high $\mathrm{Var}(b_t)$ show signs that the trailing pole could have made contact with the surface, but

did not stick for more than a few seconds (example: green highlight). We think it is sensible to call this behaviour walking because the horizontal state is not maintained. We did not investigate this type of event further because of the small sample size and because we have no way to independently verify whether the trailing pole made contact with the surface.

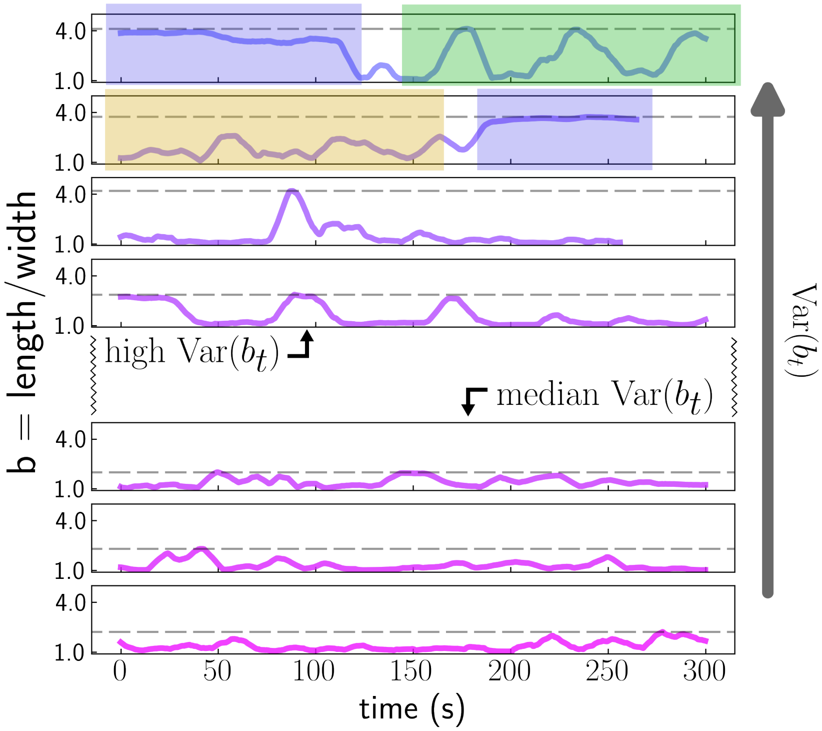

Figure 3: Example coarse-grained aspect ratio time series for several walking trajectories, sorted by decreasing Var(b_t). Blue highlighted regions are suspected crawling behaviour, yellow highlighted region is typical walking behaviour, variations in the aspect ratio for green highlighted region are large enough that the trailing pole might make brief contact with the surface, but otherwise this region is similar to walking behaviour.
