# Supplementary material for: Data–driven modelling makes quantitative predictions regarding bacteria surface motility
Source: PLoS Comput Biol. 2024 May 14;20(5):e1012063. doi: 10.1371/journal.pcbi.1012063 (PMC11125545; doi:10.1371/journal.pcbi.1012063)
Supplement: Attachment — Submitted filename: Twitching_Motility-Response-Lett.pdf [file pcbi.1012063.s012.pdf]

PCOMPBIOL-D-22-01843:

Data–driven modelling makes quantitative predictions regarding bacteria surface motility

Reply to the referee comments

Daniel Barton, Yow-Ren Chang, William Ducker, Jure Dobnikar

June 3, 2023

We thank the referees and the editor for their efforts in reviewing our manuscript and for their constructive criticisms. We carefully addressed all the comments made by the referees and in response to them we significantly improved the manuscript. Following the advice from the referees, we added a few new figures, performed additional analysis, and clarified several parts of the text that might have been less clear in the original submission. While addressing a question posed by both referees, we became aware of experiments of Zhang et al. measuring the reduced retraction speed of TFP under conditions closely matching those from the tracking data we are using for the analysis. It turns out that these experiments provide a direct support for our conclusions that the retraction speed of TFP (under these conditions) is much lower than in several other experiments previously reported in literature. We have highlighted this at several places in the manuscript and we believe that the supporting experimental results make our paper much stronger.

We hope that our revised manuscript is now suitable for publication in PLoS Computational Biology. Below, we provide our detailed response to the referee comments.

Reviewer 1

The manuscript by Barton et al uses computational model for twitching motility of P.aeruginosa bacteria in combination with trajectory analysis and Bayesian inference with a goal of gaining access to the parameters that are not otherwise easily accessible in experiments. We find the idea certainly useful and approach generally promising. The manuscript is mostly clearly written and introduces all the details of simulations, data analysis, and statistics rather well. Introduction and motivation of the study is also convincing. We noticed, however, that the realisation of the approach developed in the ms was hampered by several deficiencies in the study.

i) First conceptual point is that the manuscript going in the direction of the method suggestion have chosen the experimental system that does not allow to validate the proposed approach. It is true that many parameters that the authors try to restore with the help of the model still can't be measured, but the pili dynamics for the bacteria species they are using can be visualised (and was visualised before, starting from the seminal paper of Skerker and Berg). For sure that would be much shorter time series than tracking data but that would be the crucial experimental data to validate the performance of the model simulations (which does have some limitations and apparent discrepancy with previous observations, see next point)

Reply: We appreciate that the referee evaluates that our manuscript is "going in the direction of method suggestion", as it reflects the broader applicability of the method we developed. We agree that a similar data driven approach would be useful in many other systems, however, we also do think that it is well-applicable to study the surface motility of bacteria and that our analysis of *P. aeruginosa* twitching has resulted in a number of novel and non-trivial conclusions.

Regarding the comment on extracting parameters from various experiments, we would like to point out there is evidence that the properties of bacterial twitching depend on the bacteria species (actually even on the exact strain of bacteria), and on the environmental conditions, which makes it difficult to quantitatively compare observations in different experiments. In particular, the experiments in the Skerker & Berg paper were performed on a different strain of *P. aeruginosa* than the experiments of Jin et al., from where we obtained the tracking datasets. While we assume that the physical model we introduced is general enough to describe twitching in these different conditions, we do not expect the parameter values to be the same. Moreover, Skerker & Berg published a few very short movies of twitching with a relatively coarse time and space resolution, from which it would be almost impossible to reliably extract the microscopic parameters for our model. In general, raw data from observations of bacterial motility is rarely published and we have not been able to identify an experimental lab who could provide us with long time tracking data combined with dynamic TFP imaging of the same system at the same conditions. We therefore took a conservative approach and fixed only those parameters that have been determined accurately and reliably in independent experiments. For the rest, we perform the sensitivity analysis to assess the influence of each parameter on the statistical properties of the trajectories, identify the essential parameters and build up a minimal model, which allows us to obtain estimates by performing Bayesian computation.

A project that collects *P. aeruginosa* tracking data from as many sources as possible and performs a standardised analysis on them would be extremely valuable, however, this is beyond the scope of our current manuscript.

ii) We believe that the shortcomings on the model side might be the true reasons why the proposed parameter optimisation approach could not excel in fixing all of the parameters. Several indications that the model was not yet properly tuned include the suggestion of authors that motility is driven by short displacements of multiple pili (which seems to contradict the observations from the same Skerker and Berg paper and pili-driven motility as was imaged for Neisseria gonorrhoeae)...

Reply: Indeed, it seems surprising that our model predicts relatively short median displacements by individual TFP – in contrast to the movies of Skerker and Berg where individual TFP were seen to move the bacteria by micrometer scale distances. In response to the comments by the referee, we have carefully analysed all the trajectories available from the tracking experiments of Jin et al.

and searched for evidence of such longer displacements. Since the experimental trajectories have significant measurement noise, this data is typically pre-processed to obtain smooth trajectories for further analysis. This pre-processing results in a better signal to noise ratio but in turn makes it difficult to extract evidence of individual TFP processes from the tracking data. We developed a general algorithm for extracting piecewise linear features from unprocessed noisy trajectory data using an estimate for the scale of the spatial error in the tracking data to adjust its sensitivity (unpublished research). A part of a typical experimental crawling trajectory and its piecewise linear transformation are shown in Figure 1. The algorithm consistently finds statistically significant linear segments that are much smaller than $1\ \mu m$; which we can also verify by eye. Figure 1b shows the length distribution of the piecewise linear segments, which are typically much less than one micrometer. We performed the same analysis on all trajectories we used in the analysis and conclude the TFP of this particular *P. aeruginosa* strain in the particular experiment typically do not generate micrometer sized displacements. We do not think that our observation is necessarily in conflict with the results

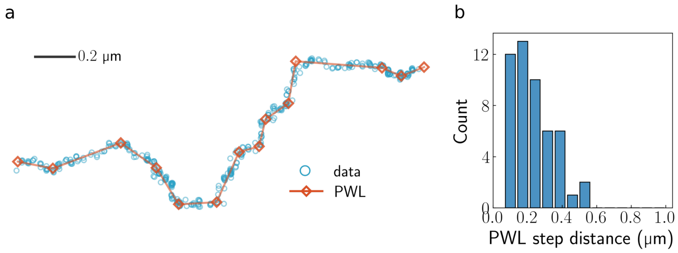

Figure 1: (a) Noisy measurement data for a 40 s duration segment of a typical crawling trajectory (blue circles), (a) The piece-wise linear approximation method provides insight into the typical size of displacements generated by individual TFP. (b) Piecewise linear (PWL) step distance distribution for the full duration (105 s) of the trajectory in a). The method detected 50 linear segments, with a median length of $0.21\ \mu m$ and a maximum length of $0.55\ \mu m$.

of Skerker & Berg because (i) the strains and experimental procedures used in these two experiments are different, and (ii) it is not clear to what extent Skerker and Berg can resolve sub-micron displacements, nor is that a theme they develop in their work [1]. The same two points apply to more recent direct observations of twitching *P. aeruginosa* [2].

ii)... Second point, specific to PA cells, is that in the model the cells can only move one way (walking or crawling) depending if potential is switched to attraction or repulsion while in the experiments [7] it was shown that individual cells can switch between the modes.

Reply: We agree with the referee that in general, the bacteria can transition between walking and crawling modes, however, these transitions turn out to be rare in the data we worked with. We thus deliberately focused on analysing the two motility modes separately since this approach allows us to put aside some of the details of the body–surface interaction in our analysis.

We analyse variations in the aspect ratio of the cell bodies, $b = \text{length}/\text{width}$. At the original 0.1 s resolution of the experiment, there is measurement noise in the value of the aspect ratio, so we coarse grained it using a sliding window of 20 s (200 frames), call the coarse grained time series b_t with minimum value $b_{\text{min}} = \min(b_t)$. We expect most walking trajectories to have b_{min} close to

1 because they eventually pass through a nearly vertical state during twitching. In the paper, we equate trajectories with $b_{\text{min}} < 1.6$ as walking and those with $b_{\text{min}} > 1.6$ as crawling. The $b_{\text{min}} < 1.6$ threshold selects 371 out of 3113 trajectories that exhibit some walking behaviour. We also select 100 trajectories with similar mean velocities from the $b_{\text{min}} > 1.6$ (crawling) subset and check them by eye for possible transitions between walking and crawling or other aberrations. A small handful of trajectories appear to transition between walking and crawling states, which we removed and recorded in a separate list. From the remaining trajectories, we manually filtered out 175/371 walking and 63/100 crawling trajectories and used these subsets for our analysis.

We plotted the aspect ratio (b_t) time series to evaluate whether the trajectories in the crawling and walking subsets are correctly identified. For convenience, we sort the trajectories by the variance of the aspect ratio, $\text{Var}(b_t)$, with the idea that trajectories that transition between walking and crawling will have high variance. Figure 2 shows the distributions of $\text{Var}(b_t)$ for the 175 walking trajectories and 63 crawling trajectories. Aspect ratio profiles for trajectories in the crawling data set with median $\text{Var}(b_t)$ and max $\text{Var}(b_t)$ are shown in Figure 3. Like most crawling trajectories, the median trajectory has a fairly constant aspect ratio while the other shows some indications that it makes out-of-plane rotations for a small part of its duration. Further purifying the collection of crawling trajectories by removing a few trajectories with high $\text{Var}(b_t)$, does not significantly affect our results.

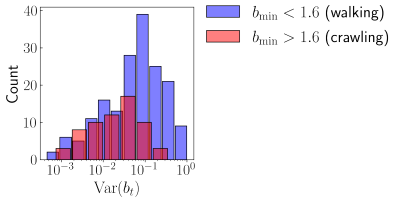

Figure 2: Distributions of the variance of the aspect ratio $b = \text{length/width}$, which is coarse grained using a sliding window of 10 s (100 frames), call this time series b_t. In the paper, we separate trajectories using a threshold of $b_{\text{min}} = 1.6$ where b_{min} is the minimum of the coarse grained aspect ratio.

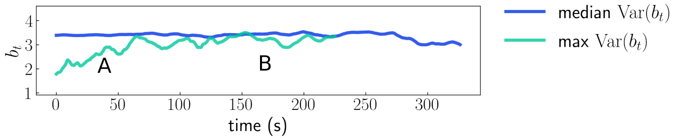

Figure 3: Coarse grained aspect ratio time series for the median $\text{Var}(b_t)$ trajectory and the the maximum $\text{Var}(b_t)$ trajectory. The latter trajectory may be in the walking state for the first $\sim$50 s (marker A) and then transition to the crawling state (marker B). The variations in b_t in the vicinity of marker B might be due to small out-of-plane movements, or they could be simply be due to the tracking algorithm. Removing a few trajectories with high $\text{Var}(b_t)$ from the crawling subset does not significantly affect our results.

Figure 4 shows examples of the aspect ratio time series for walking trajectories, sorted by $\text{Var}(b_t)$. The top half of the figure shows the four time series with the largest $\text{Var}(b_t)$ (where necessary, time

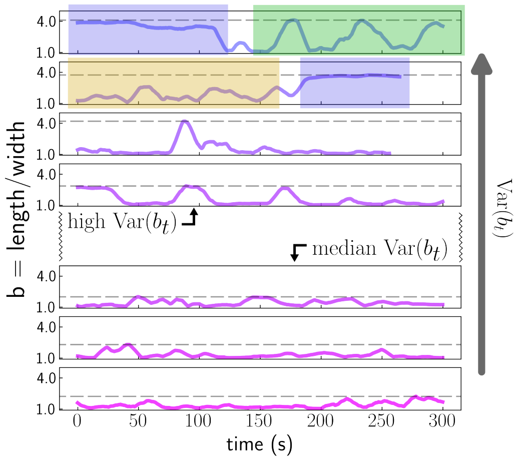

Figure 4: Example coarse-grained aspect ratio time series for several walking trajectories, sorted by decreasing $\mathrm{Var}(b_t)$. Blue highlighted regions are suspected crawling behaviour, yellow highlighted region is typical walking behaviour, variations in the aspect ratio for green highlighted region are large enough that the trailing pole might make brief contact with the surface, but otherwise this region is similar to walking behaviour.

series are truncated to 300 s so that they fit in the figure.) The bottom half shows more typical walking trajectories. We are not concerned that a very small number of trajectories with high $\mathrm{Var}(b_t)$ show evidence of partial crawling behaviour (regions highlighted in blue) because we checked that removing a few such trajectories from the subset does not significantly affect our results. Interestingly, a few trajectories with high $\mathrm{Var}(b_t)$ show signs that the trailing pole could have made contact with the surface, but did not stick for more than a few seconds (example: green highlight). We think it is sensible to call this behaviour walking because the horizontal state is not maintained. We did not investigate this type of event further because of the small sample size and because we have no way to independently verify whether the trailing pole made contact with the surface.

Action: We included the above discussion in revised Online SI (*S9: Aspect ratio profiles*).

The rest of the work, in particular the statistical analysis and discussion of the parameters and why some of them can't be too efficiently optimised etc. is thoroughly written.

Below we specify more concrete points of criticism, in chronological order as they appear in the text, and hoping the authors would be able to respond to those.

1) typo: in introduction, NG name should be italic

Reply: We thank the referee for spotting this. We have corrected the typo.

2) minor: page 5, before section A. The orientation of the cell in the model is regulated by the attraction/repulsion of the two point on the cell body. While it is clear why that is needed for the

model, what is the biological explanation/basis to introduce such interaction potential?

Reply: The bacteria surface interaction during twitching is difficult to measure and not much is reported about it. To avoid making unverifiable assumptions about the interaction, we study walking and crawling separately for the majority of this manuscript. A purely repulsive interaction is used to study the walking state, while a sharp potential well is used to study the crawling state by effectively constraining the z-coordinate of both poles of the crawling bacteria in a plane parallel to the x–y plane. We argue that for a spherocylindrical bacteria on a planar surface, two points of contact provide a simple and reasonable approximation of the un-sticking of the body from the surface. In the section V C. on transitions between walking and crawling we choose a softer surface interaction so that the surface forces become comparable to the TFP mediated forces and the transitions in the simulations are possible.

Action: We rewrote parts of the section on the interaction potentials in the revised Online SI to improve the clarity of the model description (*S1: Surface Interaction*)

In the revised manuscript, we added the following text on Page 5 (section II. Modeling):

"... A result of this torque is that a significant attractive body–surface interaction is necessary to maintain the crawling state. The majority of the bacteria in the experimental tracking data that we analyse [3] are crawling for the entire duration of the measurement, which suggests these bacteria stick firmly to the surface. Skerker & Berg also argue that the surface interaction between *P. aeruginosa* and the glass coverslip must be quite strong in their experiment [1], because many of the bacteria they observed did not move, despite showing TFP activity. Since bacteria in this data set rarely transition between crawling and walking states (see S9 Appendix), we deliberately study crawling and walking behaviour separately both in simulations and experiment until section **??**.

In order to simulate both walking and crawling modes of motion, the cells are decorated with two interaction sites (one at each pole, see Figure 1b). The short range interaction of the two sites with the surface is either purely repulsive, or an attractive well (see S1 Appendix for details). A purely repulsive surface interaction is used to simulate walking motility while crawling motility is simulated by initialising both poles in contact with the surface and using a strong attractive potential to maintain the horizontal orientation of the cell. This approach avoids the need for more detailed modelling of the bacteria-surface interactions which are poorly understood and may vary between experiments and between individual bacteria. "

3) minor: throughout the text the quantities are inconsistently given either with units or without, while obviously they should be always with units (if they are dimensional)

Reply: Thank you for pointing this out. We revised the text and made this consistent.

4) Major: the model is solved by energy minimisation algorithm thus disregarding the dynamics happening at short time scales. This dynamics is influenced by the viscosity of the surrounding solvent and friction with the substrate. While the authors mention that their model can reproduce the slingshot movement in their model that would correspond to immediate cell body equilibration (jump) while in reality it would be a viscosity damped process. Friction forces were suggested to be important also in the context of twitching motility (see Pönisch et al PRE 2109), while it can be

absorbed in effective viscosity for purely 2D motility, in this work, 3D effects and forces in z-direction are obvious and would affect the friction force and thus the dynamics. With current approach of simple energy minimisation, those are hard to follow.

Reply: Based on this comment, we expanded our discussion on the subject of the relative influence of the forces in the system. Our assumption is that viscous forces are much less than friction forces which are less than the maximum forces generated by TFP. Although we are aware of some experiments which use AFM to measure surface interaction forces by measuring the force needed to separate a bacterium from a surface, to our knowledge, the friction forces of *P. aeruginosa* bacteria during twitching have not been adequately measured. Therefore each part of this assumption needs to be argued.

Viscous forces: Pönisch et al. (2019) estimate the viscous forces to be approximately $0.2\ \mathrm{pN}$ [4] for *N. gonorrhoeae*. Walking *P. aeruginosa* bacteria make out-of-plane rotations which may lead to more significant viscous forces, but it is still reasonable to assume that the system is dominated by TFP forces in most cases. In addition, since crawling is the dominant motility type in this tracking experiment, we are primarily concerned with an accurate simulation of that mode.

Friction forces: Skerker & Berg argue that the interaction between their *P. aeruginosa* and the glass cover-slip is relatively strong, because many of their bacteria did not move despite showing TFP activity. We also see individual bacteria in the tracking data that do not move. One interpretation is that the individual bacteria have a range of surface interaction strengths (which could depend on the expression of adhesive proteins). By focusing on crawling bacteria, we may have selected a subset of the population with the appropriate surface interaction strength for this motility type. Likewise, the walking bacteria subset may be those with a weaker surface interaction strength. As a parallel argument, in section V.C we demonstrate using our model that a strong surface interaction is necessary for crawling behaviour because of the way TFP pulling forces generate a torque on the cell in this geometry. Although in that case, the force is acting to pull the trailing pole off the surface (un-sticking force), instead of dragging the body across the surface (friction force), we argue these forces should be proportional to some extent. The model TFP have a consistent retraction velocity which we interpret to be the typical retraction velocity under the loads that arise from friction with the surface. The retraction velocity of *P. aeruginosa* TFP has been shown to vary under load by [5] and [6]. Since our approach to modelling is data-driven, and there is no additional data available about the friction force in the experiments we study, we don't make more complex assumptions about how the friction force influences the dynamics. See also our response to comment (5) regarding pili retraction speed.

Action: In the revised manuscript, Page 6, section II. Model, we added the following text to discuss the treatment of dissipative forces in our model:

"The force needed for crawling bacteria to maintain their horizontal orientation is one property of the interaction between the bacteria and the surface. A related property, which we do not model explicitly, is the surface friction force. We expect that the primary effect of friction is either to cause the molecular motor to work at a higher power or to reduce the retraction speed of TFP. In the former case it has no effect on the trajectories, and in the latter case it renormalizes the TFP retraction speed. The idea that the retraction speed of *P. aeruginosa* TFP is reduced by the tension incurred from dragging the cell is supported by both direct observation of twitching [6, 1] and single pilus

experiments [5]. Although we are not aware of precise measurements of the friction forces between the bacterium and surface during twitching, we argue that for both walking and crawling bacteria, the maximum force generated by a pilus is larger than the friction force, otherwise these bacteria would hardly move. In addition, like Pönisch et al. [4], we neglect viscous forces based on the assumption that they are small compared to forces generated by the TFP motor, and at least in the case of crawling bacteria, we assume viscous forces are small compared to surface friction forces as well."

Slingshots: We do not discuss slingshots as described by Jin et al. (2011) [7] in detail here. This is partly because a computer model has already been reported that reproduces slingshot-like behaviour [8] and partly because we were not able to reproduce all of the results of Jin et al. (2011) using the data analysis they describe. To be clear, we also see a strong correlation between high velocity displacements and rotations of the cell body in the experimental data we study and in simulations. Simulations show that this behaviour often corresponds to the detachment of a pilus from the surface. When we mention slingshot behaviour in our discussion, we are referring to this correlation. Where our data differs from Jin et al. is that we could not find experimental trajectories for which the distribution of velocities of the leading pole is bimodal (in linear or log-scales), forcing us to come up with alternative definitions of slingshot events based on both the velocity and direction of motion. Subsequently, we find that slingshots rarely make up a large fraction of the total contour length of the trajectory for the bacteria in this tracking data.

5) Major: pili retraction velocity and pili detachment rate were experimentally measured for Tfp and were shown to play central role for describing twitching motility (see Marathe et al Nat. Com. 2014 and Zaburdaev et al Biophys. J. 2014). The authors had to reduce the retraction speed (by hand so to say) to make their model produce a better fit to the data. In fact, as the attachment/detachment rates are the central parameters of the model, we feel that disregarding these effects is one of the major reasons why the model fails to give better alignment to the data (meaning more convincing matching by statistical analysis).

Reply:

Pili retraction velocity: Skerker and Berg measured the retraction velocity of unloaded TFP for the PAK strain of *P. aeruginosa* to be approximately $0.5\ \mu\mathrm{m/s}$ [1] although they note that they expect retraction to be slower under load. This estimate is consistent with $0.64\ \mu\mathrm{m/s}$ measured by Koch et al. (2021) [9] (PA01 strain), and broadly consistent with retraction velocities measured for *N. gonorrhoeae* [10, 11]. Ribbe et al. (2017) measure the retraction velocity of pili for the PA14 strain of *P. aeruginosa* [5]. They measure average retraction velocities of slightly more than $1.1\ \mu\mathrm{m/s}$ under an 8 pN load and note the change in temperature from 29° (Skerker & Berg) to 37° as a reason for the $2\times$ faster retraction rate [5]. Notably, the retraction velocity does fall quite sharply as the load increases in that experiment, suggesting a core difference between *P. aeruginosa* and *N. gonorrhoeae*. Despite the variety of bacteria strains and experimental procedures represented, we originally interpreted these results as a consensus on the average pili retraction velocity of between 0.5 and $1.0\ \mu\mathrm{m/s}$ and concluded that this would be a good estimate for the PA01 *P. aeruginosa* in the data that we study. Although unknown to us at the time of submission, Zhang et al. (2021) measure the retraction velocity of surface attached TFP to be $0.09\ \mu\mathrm{m/s}$ and unbound TFP to be $0.17\ \mu\mathrm{m/s}$ [6] for (PA01) *P. aeruginosa* in very similar experimental conditions to our data. They argue that the bacterial strain (PA01 vs PAK) is the most likely reason for the discrepancy between their results

and Skerker & Berg. We studied all of the experimental procedures described in the papers cited here and did not simply take their published results at face value. Our conclusion is that all of these authors most likely give accurate estimates of the pili retraction velocity *in their experiments*, but that this value is actually quite sensitive to a number of environmental factors as well as the specific strain of the bacteria. If we take only measurements on PA01 *P. aeruginosa* bacteria, and only direct observations of TFP that are under load from pulling the cell on the surface, then the most relevant estimate is certainly Zhang et al [6] which is $0.09\ \mu m/s$. For reference, our estimate on page 20 of the original draft is $0.132\ \mu m$ with a 90% confidence interval of $[0.074, 0.20]\ \mu m/s$.

Action: Due to the referee comments in the first round, we became aware of the experiments of Zhang et al. that directly support our conclusions, we revised our discussions of the retraction speed at a few places in the manuscript. In the revised manuscript we now write:

Abstract: "...We note that the motility mechanism is highly sensitive to the experimental conditions and predict an effective retraction speed for TFP, which is much smaller than some previously reported values obtained in other experiments...

II A. Kinetic Monte Carlo Simulations, Page 8: "...The second possibility is that the retraction speeds measured in certain other experiments are simply not accurate or relevant for the tracking data that we study, given that the experimental procedures and conditions vary in one or more important aspects. To study this possibility, we fix $\alpha = \pi/2$ and make $v_{\rm ret}$ a variable in the model. Using the method described in section V, we predict an average retraction speed $v_{\rm ret} = 0.13\ \mu m s^{-1}$, which is strongly supported by the recent experimental measurement of Zhang et al. [6]."

V A. Parameter inference for crawling trajectories: "... This value implies that, in most configurations, TFP can only make small and inefficient retractions with small contribution to the trajectory. Such a strong dependence of retraction angle on the retraction speed is non-intuitive and has not been confirmed in experiments. Therefore, we decided to probe whether the retraction speed itself, despite being reported consistently several times [1, 9], might not be accurate for our tracking data. We stress that the different measurements of $v_{\rm ret}$ were not performed at the same conditions, but at different strains, temperatures, and preparation protocols. Moreover, a recent measurement [6] was reported while we were developing our results, which predicts a significantly lower value than the previous studies. The experiments [6] are performed at the same conditions and with the same protocol as the tracking data we use. Therefore, we also explored the retraction speed itself as a variable parameter and predicted its value from tracking data. To do this, we set $\alpha = \pi/2$ and let the retraction speed vary in the range $[0.05, 1.0]\ \mu m s^{-1}$. The approximate Bayesian computation was then repeated with four parameters, (see S4 Appendix). The shape of the new posterior is very similar to that in Figure 5, and we estimate $\bar{v}_{\rm ret}$ to be $0.132\,[0.074, 0.20]\ \mu m s^{-1}$. Our estimate is consistent with the experimental value of Zhang et al. [6], which is $0.09\,\mu m s^{-1}$. Zhang et al. argue that the difference between their measurement and that of Skerker & Berg is due to the use of different strains of *P. aeruginosa* (PA01 vs. PAK). Other previously reported values have been obtained at different temperatures (37° [2, 9, 5] vs. 30° [6, 1]). Further, Koch et al. [9] measure the retraction speed of TFP which are not interacting with the surface and are therefore not renormalized by friction forces. We conclude that the experimental observation [6], which is most relevant to the tracking data we study [3], fully supports this prediction. Hence, we argue that the renormalised retraction speed is a more plausible explanation for the effect of friction forces on the motility than the angle dependent retraction speed that we studied initially."

VI. Discussion: "This analysis yields an estimate for the retraction speed of TFP that are actively pulling the cell: $v_{\rm ret} \approx 0.13\ \mu{\rm ms}^{-1}$, which is in good agreement with the most relevant experimental measurement that reports the retraction speed of $0.09\ \mu{\rm ms}^{-1}$ for loaded, and $0.17\ \mu{\rm ms}^{-1}$ for unloaded TFP [6]."

Pili detachment rate: Tala et al. measure the median surface attachment time of pili of PA01 *P. aeruginosa* to be 1 s (ΔfliC) and 2.3 s (wild-type). Although the unexplained discrepancy between ΔfliC and wild-type is curious, we think these measurements of the pili detachment rate are more relevant to our work than measurements on *N. gonorrhoeae*. Especially considering that it is unclear to what extent the tug-of-war mechanism used to explain the movement of *N. gonorrhoeae* can be applied to *P. aeruginosa*. The tug-of-war mechanism explains how *N. gonorrhoeae* achieves persistent motion despite having TFP distributed all over its body, on the other hand, *P. aeruginosa* achieves persistent motion by a combination of localising its TFP on one pole and sticking its body to the surface to support the crawling state. Tala et al. report surface sensing and catch-bond like mechanisms to explain their observations of TFP surface attachment and retraction in *P. aeruginosa* bacteria, and while we do not think that any single experiment is definitive, we choose to focus on this behaviour in our modelling.

6) minor/Major: related to the above, strangely the pili pulling force or stalling force is actually never a parameter of the model, while biophysically this is the major quantity determining the dynamics.

Reply: In response to this comment, we ran the sensitivity analysis again and included the TFP stall force as a variable parameter. The analysis confirms our intuition that the value of the stall force does not have a strong influence on the simulated trajectories. Due to a lack of good experimental data on the topic, we do not explicitly model the friction forces in the system, instead we assume that friction forces (and viscous forces) in the system are dominated by the TFP mediated forces. As a result, varying the stall force in this model does not lead to qualitatively different motility patterns. When we calibrate the model against experimental data, we predict the TFP retraction speed to be approximately $0.13\ \mu{\rm ms}^{-1}$. This is our estimate for the retraction speed under the typical loads that arise from dragging the cell across the surface. The fact that this number is supported by experimental measurements is good evidence in support of the model [6].

Action: In the revised manuscript, we include the sensitivity analysis for the stall force (Table III), and added the following text (IV B. Deriving a minimal model using sensitivity analysis, Page 17): "As discussed in section II, a core assumption in this model is that the TFP stall force typically dominates the friction and viscous forces in the system. The stall force $f_{\rm stall}$ and the elastic modulus E still play a role in the dynamics in principle by determining the maximum extension that a pilus can support relative to its equilibrium length before the motor is stalled. However, as demonstrated in Table **??**, the simulated trajectories are not sensitive to these two parameters and varying them does not lead to qualitatively different motility patterns."

7) minor: explanation of what the free pili does during interaction with a surface (bottom of page 7) - is not clear and should be rewritten

Reply: We agree that the original text was less than ideal and we re-wrote this part of the text, which is now hopefully more clear. We shifted this paragraph to the end of the subsection, because it describes a minor implementation detail.

Action: In the revised manuscript (section II., Page 9), the text now reads: "Detachment transitions are accompanied by shrinking of the pilus by one unit of 4 nm. For taut pili, shrinking by 4 nm is enough to ensure that after detachment the pilus does not intersects the surface anymore. If the pilus is not taut at the moment we attempt to detach it, then it may intersect the surface after detachment and re-attach at the end of the simulation step. In this case, we repeat the shortening until it no longer intersects."

8) Major: result of the model saying that motility of cells is a result of multiple small displacements by multiple pili is not consistent with previous images/movies of twitching motility and is not critically commented in this respect.

Reply: Although the model predicts some large displacements, it also predicts large numbers of very small displacements. As in the response to the comment (ii), we used an unpublished algorithm to extract piecewise-linear features from the trajectory data and discovered statistically significant linear features in the data much smaller than those usually reported. We do not think that this necessarily contradicts other reports, because various experiments can only report displacements of bacteria on scales that they can in-fact resolve. Consistent with our response to comment (5), we think that it is useful to compare our results with the results of experiments using different bacteria, different strains of *P. aeruginosa* and different experimental conditions and procedures, however, such comparisons should be made conservatively and quantitative agreement should not be the default expectation. The numerous small displacements predicted by our model are almost all generated by TFP that are anchored to the surface-facing side of the cell where they can make contact with the surface after a short extension, and drive the forward displacement of the cell by a short retraction. If such short TFP exist and contribute the motion of the cell, not only would these pili often be below the detection resolution of current experiments [2], but they may also be occluded by the cell body.

9) Major: Section C on page 23 says that the cells in the model either crawl or walk and can't switch their behaviour in the model, referring to [7], but also in [7] it was shown that individual cells can switch the modes of activity. So how that would be captured/explained by the model.

Reply: In this model transitions between walking and crawling states depend on the body–surface interaction strength and the distribution of TFP, as we show in section V C. Since we do not have experimental measurements for the body–surface interaction, we deliberately extracted two subsets of experimental trajectories from the tracking data: one subset exhibiting purely crawling and one subset exhibiting purely walking behaviour. In the Bayesian analysis, we compare the crawling subset to simulations with strong surface attraction, and walking subset to simulations with surface repulsion. This allows to calculate optimal model parameters separately for the walking and crawling motility that we see in our tracking data. In doing so we remove one parameter, the surface interaction strength, from consideration. In section V C, we reduce the strength of surface attraction to a value that makes it comparable to the TFP generated forces and study transitions between states.

10) Minor: In the Appendix formula explaining body equilibration it seems that also shortening of the pilus would generate a force, while it says the opposite in the text. So maybe authors should comment on it.

Reply: Model TFP cannot push the cell by extension, we amended the supplementary information to make this clear.

Taken together, we think that model needs to be critically reassessed by the authors in the context of known pili-substrate interactions, force dependent detachment and retraction velocity, as well as the critical discussions of the observed motility with previously reported cases. Thus we consider the ms as not yet suitable for publication.

Reply: We hope that our response to all the constructive comments, and the improvements that we have made in the revision, now make the manuscript suitable for publication.

Referee 2

In the present manuscript, Barton et al. first set up a model for twitching motility of bacteria at surfaces, driven by retraction and extension of type-IV-pili. The model, which is based on experimental observations of this type of systems, incorporates the stochastic growth and shrinkage of pili, their reformation as well as their random distribution at one cap of the bacterium, which is modeled as a spherocylinder. Depending on the interaction potential of bacterium and surface, two modes of motion can be described: walking and crawling. In the second part of the manuscript, a sensitivity analysis for the model parameters is performed and the most sensitive four model parameters are identified (the others are kept fixed). In the final part of the paper, the most sensitive model parameters are inferred based on tracking data of the bacterium P. aeruginosa by approximate Bayesian computation.

The authors set up the model in a very systematic way. The presentation of is in all parts clear and comprehensible. It is particularly noteworthy that parameters are systematically inferred from experimental tracking data. In this context, the authors go beyond standard approaches of fitting mean-square displacements and (velocity-) correlation functions. I therefore support the publication of this manuscript in PLOS Computational Biology.

I would like the authors, however, to take the following questions and comments into account in their revision prior to publication.

(I) trajectories, correlation functions and MSD

Though the parameter inference does not rely on standard measure, I would still encourage the authors to include those in the manuscript. First, some exemplary experimental trajectories could be shown in comparison to the simulated ones. This will help gaining some intuition on the type of random transport process. Furthermore, a comparison of simulated and experimentally obtained mean-square displacement could now be used to compare the success/goodness of the modeling and parameter inference.

Reply: We thank the reviewer for the invitation to expand the data analysis portion of our manuscript. We added a panel showing example mean squared displacement curves for several typical walking and crawling trajectories (Figure 5 in this document / Figure 3 in the revised manuscript). The mean squared displacement scaling exponent, k, is usually close to 1 for walking trajectories and is

much larger for crawling trajectories. The second panel of Figure 5 are the population distributions of k for the walking and crawling trajectories that we study in this manuscript. An additional figure has been added which shows several simulated and experimental trajectories that have statistically similar properties (Figure 6 in this document). The experimental trajectories are typical crawling trajectories and the simulated trajectories are obtained by calibrating simulation parameters against experimental data using the method described in the parameter inference portion of the manuscript.

We already briefly discuss MSD as a measure of the quality of the model in the results section of the manuscript. When we calibrate the model against crawling trajectory data using a set of statistics that does not include the mean squared displacement, we find the mean squared displacement also matches well.

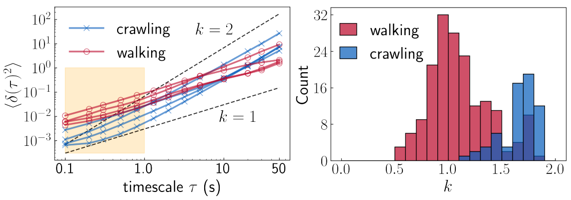

Figure 5: Sample MSD curves and k distribution. Shaded region is typically dominated by measurement noise.

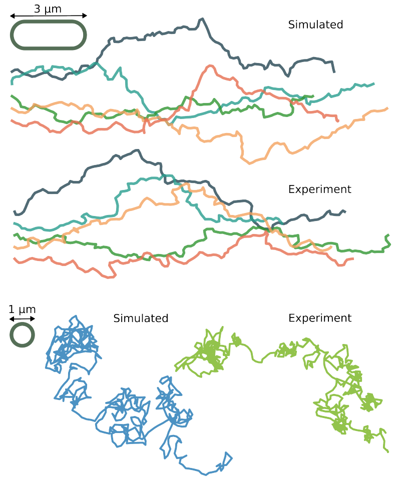

Figure 6: Example simulated and experimental crawling trajectories. Crawling trajectories are truncated to the first (330 s) and rotated to align with the horizontal axis. A spherocylinder with length 3 µm and width 1 µm is drawn for scale. The simulated walking trajectory is truncated to the first 1000 s and the experimental walking trajectory is 870 s in duration.

Action: In the revised manuscript, we added panels d) and e) to Figure 3, and we introduced a new Figure 4 showing the example experimental and matching simulation trajectories.

We also added the following paragraph: "Given a set of experimental trajectories, the next section deals with calibrating the model to produce simulated trajectories that have similar statistics. Before developing a set of statistical tools to accomplish this, it is helpful to look at some example simulated trajectories to see that the model can produce trajectories with statistics that are, at a glance, visually indistinguishable from real twitching trajectories, see Figure 6. "

(II) trail following

There have been works, for example by Ramin Golestanian and others, discussion the question of trail following in the context of twitching motility, in particular in the context of P. aeruginosa. The model presented by the authors does not include this effect. Please clarify whether this mechanism is relevant for the understanding of the (long-time) motility of these bacteria and why it was left out in this study.

Reply: One major difference that we see between trail following experiments and the experiments that we study is the experiment duration. Trail following experiments typically last many hours, in contrast, Jin et al. introduce the bacteria to the surface, wait 15 minutes for them to attach, and then record the surface motility for another 30 minutes. We expect the build up of Psl or other excreted chemicals is not significant in this time frame. In response to the reviewer comment, we decided to examine this assumption more carefully.

To do this we reconstructed the field of view of the microscope (Figure 7). Supposing that bacteria deposit Psl on the surface at their location, for simplicity we draw a circle with radius $R = 0.5\ \mu\mathrm{m}$ at the position of the leading pole of each bacteria and increment the pixel values in these regions. We then follow the time evolution of the trajectories and repeat the process of incrementing pixel values on each frame. This procedure gives us an estimate of where the Psl trails would be. We then follow the time evolution of each trajectory individually and compute its overlap with all the other trails. If A is the number of pixels in a given trail, and B the number of pixels in that trail that overlap with other trails, then we define the overlap fraction as B/A.

From Fig. 7a, we see that trajectories have a variety of overlap fractions. To understand whether such overlaps influence the motility characteristics, we look for correlations between overlap fraction the summary statistics that we use to analyse trajectory data. The statistics used are the mean velocity, the variance of the deviation angle, the persistence and the activity. We see no correlation between the overlap fraction and the first two statistics, while the persistence and activity statistics are slightly correlated with overlap fraction only at very fractions. This slight correlation could be due to a build of Psl or it could due to some other surface adaptation process. A detailed study of the way Psl trails affect *P. aeruginosa* is beyond our scope at this time since we do not have data about the surface coverage of Psl, and our analysis shows no strong influence of the trails made by these bacteria on each other. Therefore, we believe that omitting this effect from our data driven modelling is justified.

Action: We added the discussion on trail following and overlap ratio to the Online SI: *Appendix. Surface adaptation and Psl trails.*

In the revised manuscript, we added the following paragraph (section III, Page 11): "*P. aeruginosa* colonies are known to deposit extracellular polymers (EPS) on the surface, however, there is relatively little time in these experiments for a build up of EPS to significantly affect the trajectory data,

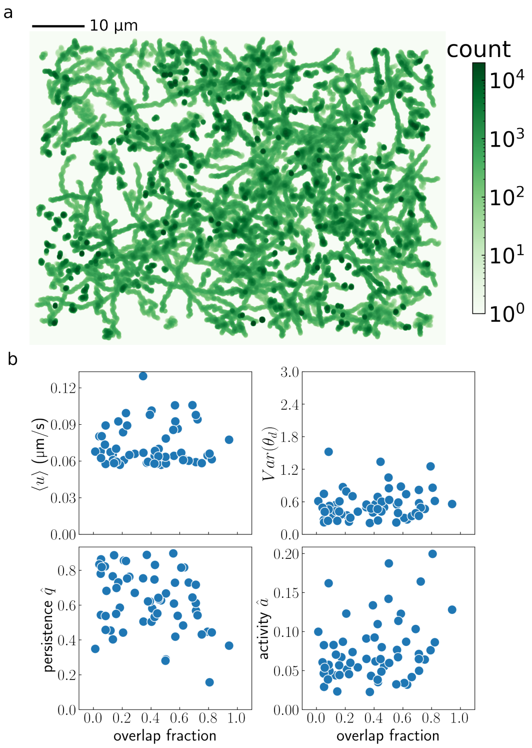

Figure 7: (a) Bacteria trails. Pixel values are the number of frames that the leading pole of the bacteria is close to that pixel. (b) Scatter plot for each summary statistic vs. overlap fraction (only for crawling trajectories, trajectories are selected using the criteria described in the manuscript.).

for evidence see S8 Appendix."

(III) simulation technique

The simulations presented by the authors rely on Monte-Carlo techniques and Boltzmann generators. These methods are designed to sample random realizations from given high dimensional distributions (oftentimes of Boltzmann type) efficiently. Depending on the specific algorithm, the resulting sequences do not necessarily reflect the temporal dynamics. In short, Monte-Carlo steps are not necessarily related to time steps. However, the comparison to experimental trajectories require that simulated data are time-ordered. Please clarify the conceptual idea of the simulation technique used here.

Reply: The kinetic Monte-Carlo algorithm that we use contains time information through the rates of the transitions between simulation states. The state vector in our model constitutes the position of the cell body and the configuration of all its TFP (including extension/retraction modes). Our simulations are sampling realizations of a continuous-time Markov chain [12], *i.e.* assuming no memory

effects. This is necessarily empirical, since the internal state of the bacteria is largely unknown.

In simulations we of course implement a discrete version of the process. For extension and retraction of TFP, we adjust the equilibrium length by a step size of 4 nm in one simulation step, this is the shortest length-scale in the system. We checked that using a smaller step size does not influence the dynamics.

We use a Boltzmann generator to generate configurations of TFP to approximately sample the distribution of TFP attachment sites on the surface. We do not consider processes such as unbound TFP interacting with each other to be crucial to model.

(IV)

There is comment (page 8) that Koch et al. made a "compatible albeit incongruous claim". This is contradicting to me and was not clear. I suggest to explain in detail what exactly is referred to here or to leave out this comment.

Reply: We recognise the sentence is not clear and leave it out. A deeper discussion of the compatibility of recently published results about TFP force sensing behaviour is not essential to this work.

(V) Typos

- There is a references to Figure 1c (page 5 in my version), which should be Figure 1b. - On the same page: "such as to balances the TFP tension..." - There are some inconsistencies in the notation in the list of references, in particular in the capitalization of journal titles.

I saw that the simulation code is made available on github. Is the trajectory data available too?

Reply: We will ensure that the trajectory data is available if the manuscript is accepted.

References

[1] J. M. Skerker and H. C. Berg, "Direct observation of extension and retraction of type iv pili," *Proceedings of the National Academy of Sciences*, vol. 98, no. 12, pp. 6901–6904, 2001.

[2] L. Talà, A. Fineberg, P. Kukura, and A. Persat, "Pseudomonas aeruginosa orchestrates twitching motility by sequential control of type iv pili movements," *Nature microbiology*, vol. 4, no. 5, pp. 774–780, 2019.

[3] L. Ni, S. Yang, R. Zhang, Z. Jin, H. Chen, J. C. Conrad, and F. Jin, "Bacteria differently deploy type-iv pili on surfaces to adapt to nutrient availability," *NPJ biofilms and microbiomes*, vol. 2, no. 1, pp. 1–10, 2016.

[4] W. Pönisch, C. A. Weber, and V. Zaburdaev, "How bacterial cells and colonies move on solid substrates," *Physical Review E*, vol. 99, no. 4, p. 042419, 2019.

[5] J. Ribbe, A. E. Baker, S. Euler, G. A. O'Toole, and B. Maier, "Role of cyclic di-gmp and exopolysaccharide in type iv pilus dynamics," *Journal of bacteriology*, vol. 199, no. 8, pp. e00859–16, 2017.

[6] J. Zhang, S. Li, T. Sun, Y. Zong, W. Zhang, and K. Zhao, "A simple, switchable pili-labelling method by plasmid-based replacement of pilin," *Environmental Microbiology*, vol. 23, no. 5, pp. 2692–2703, 2021.

[7] F. Jin, J. C. Conrad, M. L. Gibiansky, and G. C. Wong, "Bacteria use type-iv pili to slingshot on surfaces," *Proceedings of the National Academy of Sciences*, vol. 108, no. 31, pp. 12617–12622, 2011.

[8] Y. Brill-Karniely, F. Jin, G. C. Wong, D. Frenkel, and J. Dobnikar, "Emergence of complex behavior in pili-based motility in early stages of p. aeruginosa surface adaptation," *Scientific reports*, vol. 7, no. 1, pp. 1–10, 2017.

[9] M. D. Koch, C. Fei, N. S. Wingreen, J. W. Shaevitz, and Z. Gitai, "Competitive binding of independent extension and retraction motors explains the quantitative dynamics of type iv pili," *Proceedings of the National Academy of Sciences*, vol. 118, no. 8, 2021.

[10] R. Marathe, C. Meel, N. C. Schmidt, L. Dewenter, R. Kurre, L. Greune, M. Alexander Schmidt, M. J. Müller, R. Lipowsky, B. Maier, *et al.*, "Bacterial twitching motility is coordinated by a two-dimensional tug-of-war with directional memory," *Nature communications*, vol. 5, no. 1, p. 3759, 2014.

[11] V. Zaburdaev, N. Biais, M. Schmiedeberg, J. Eriksson, A.-B. Jonsson, M. P. Sheetz, and D. A. Weitz, "Uncovering the mechanism of trapping and cell orientation during neisseria gonorrhoeae twitching motility," *Biophysical journal*, vol. 107, no. 7, pp. 1523–1531, 2014.

[12] S. A. Serebrinsky, "Physical time scale in kinetic monte carlo simulations of continuous-time markov chains," *Physical Review E*, vol. 83, no. 3, p. 037701, 2011.
