# Supplementary material for: Data–driven modelling makes quantitative predictions regarding bacteria surface motility
Source: PLoS Comput Biol. 2024 May 14;20(5):e1012063. doi: 10.1371/journal.pcbi.1012063 (PMC11125545; doi:10.1371/journal.pcbi.1012063)
Supplement: Attachment — Submitted filename: report_plos_comp_2023.pdf [file pcbi.1012063.s013.pdf]

We went through the replies of the authors to our previous comments, with realization that not all of them were entirely satisfactory. We comment on them below where appropriate.

Reply: We appreciate that the referee evaluates that our manuscript is "going in the direction of method suggestion", as it reflects the broader applicability of the method we developed. We agree that a similar data driven approach would be useful in many other systems, however, we also do think that it is well-applicable to study the surface motility of bacteria and that our analysis of *P. aeruginosa* twitching has resulted in a number of novel and non-trivial conclusions.

Regarding the comment on extracting parameters from various experiments, we would like to point out there is evidence that the properties of bacterial twitching depend on the bacteria species (ac- tually even on the exact strain of bacteria), and on the environmental conditions, which makes it difficult to quantitatively compare observations in different experiments. In particular, the experi- ments in the Skerker & Berg paper were performed on a different strain of *P. aeruginosa* than the experiments of Jin et al., from where we obtained the tracking datasets. While we assume that the physical model we introduced is general enough to describe twitching in these different conditions, we do not expect the parameter values to be the same.

It is clear that various strains and conditions in biological experiments may result in large variability of outcomes. Our point was not to use previously available data, but to produce and analyze the data that allows to validate the approach.

Moreover, Skerker & Berg published a few very short movies of twitching with a relatively coarse time and space resolution, from which it would be almost impossible to reliably extract the microscopic parameters for our model. In general, raw data from observations of bacterial motility is rarely published and we have not been able to identify an experimental lab who could provide us with long time tracking data combined with dynamic TFP imaging of the same system at the same conditions. We therefore took a conservative approach and fixed only those parameters that have been determined accurately and reliably in independent experiments. For the rest, we perform the sensitivity analysis to assess the influence of each param- eter on the statistical properties of the trajectories, identify the essential parameters and build up a minimal model, which allows us to obtain estimates by performing Bayesian computation.

We mentioned Skerker & Berg publication as an example of data that would be particularly important to validate the results of this ms analysis, as they go against previous observations of twitching motility (in whatever bacteria or conditions that has been done). Moreover, those experiment were performed 20 years back and by now with modern imaging techniques both time and space resolution would be significantly higher allowing for proper type of analysis. Therefore, in our opinion, conservative approach would be to admit that the approach still needs validation in what concerns microscopic details of motility, see below.

A project that collects *P. aeruginosa* tracking data from as many sources as possible and performs a standardised analysis on them would be extremely valuable, however, this is beyond the scope of our current manuscript.

Reply: Indeed, it seems surprising that our model predicts relatively short median displacements by individual TFP – in contrast to the movies of Skerker and Berg where individual TFP were seen to move the bacteria by micrometer scale distances. In response to the comments by the referee, we have carefully analysed all the trajectories available from the tracking experiments of Jin et al. and searched for evidence of such longer displacements. Since the experimental trajectories have significant measurement noise, this data is typically pre-processed to obtain smooth trajectories for further analysis. This pre-processing results in a better signal to noise ratio but in turn makes it diffi- cult to extract evidence of individual TFP processes from the tracking data. We developed a general algorithm for extracting piecewise linear features from unprocessed noisy trajectory data using an estimate for the scale of the spatial error in the tracking data to adjust its sensitivity (unpublished research). A part of a typical experimental crawling

trajectory and its piecewise linear transforma- tion are shown in Figure 1. The algorithm consistently finds statistically significant linear segments that are much smaller than 1 µm; which we can also verify by eye. Figure 1b shows the length distribution of the piecewise linear segments, which are typically much less than one micrometer. We performed the same analysis on all trajectories we used in the analysis and conclude the TFP of this particular *P. aeruginosa* strain in the particular experiment typically do not generate micrometer sized displacements. We do not think that our observation is necessarily in conflict with the results of Skerker & Berg because (i) the strains and experimental procedures used in these two experi- ments are different, and (ii) it is not clear to what extent Skerker and Berg can resolve sub-micron displacements, nor is that a theme they develop in their work [1]. The same two points apply to more recent direct observations of twitching *P. aeruginosa* [2].

While we welcome the additional analysis, the initial assumption that one has to search for straight segments of trajectories is not really justified. If one looks at the movie of the original Skerker & Berg paper – the center of mass of the twitching cell is not traveling on the straight line at all. Actually, we doubt that it would be possible to identify the pili dynamics simply from trajectory characteristics. If only, then by the approach similar to the one advocated in this paper, via proper biophysical model of motility.

Reply: We agree with the referee that ingeneral, the bacteria can transition between walking and crawling modes, however, these transitions turn out to be rare in the data we worked with. We thus deliberately focused on analysing the two motility modes separately since this approach allows us to put aside some of the details of the body–surface interaction in our analysis. We analyse variations in the aspect ratio of the cell bodies, b = length/width. At the original 0.1 s resolution of the experiment, there is measurement noise in the value of the aspect ratio, so we coarse grained it using a sliding window of 20 s (200 frames), call the coarse grained time series b_t with minimum value $b_{min} = min(b_t)$. We expect most walking trajectories to have b_{min} close to 1 because they eventually pass through a nearly vertical state during twitching. In the paper, we equate trajectories with $b_{min} < 1.6$ as walking and those with $b_{min} > 1.6$ as crawling. The $b_{min} < 1.6$ threshold selects 371 out of 3113 trajectories that exhibit some walking behaviour. We also select 100 trajectories with similar mean velocities from the $b_{min} > 1.6$ (crawling) subset and check them by eye for possible transitions between walking and crawling or other aberrations. A small handful of trajectories appear to transition between walking and crawling states, which we removed and recorded in a separate list. From the remaining trajectories, we manually filtered out 175/371 walking and 63/100 crawling trajectories and used these subsets for our analysis.

We plotted the aspect ratio (b_t) time series to evaluate whether the trajectories in the crawling and walking subsets are correctly identified. For convenience, we sort the trajectories by the variance of the aspect ratio, Var(b_t), with the idea that trajectories that transition between walking and crawling will have high variance. Figure 2 shows the distributions of Var(b_t) for the 175 walking trajectories and 63 crawling trajectories. Aspect ratio profiles for trajectories in the crawling data set with median Var(b_t) and max Var(b_t) are shown in Figure 3. Like most crawling trajectories, the median trajectory has a fairly constant aspect ratio while the other shows some indications that it makes out-of-plane rotations for a small part of its duration. Further purifying the collection of crawling trajectories by removing a few trajectories with high Var(b_t), does not significantly affect our results. Figure 4 shows examples of the aspect ratio time series for walking trajectories, sorted by Var(b_t). The top half of the figure shows the four time series with the largest Var(b_t) (where necessary, time series are truncated to 300 s so that they fit in the figure.) The bottom half shows more typical walk- ing trajectories. We are not concerned that a very small number of trajectories with high Var(b_t) show evidence of partial crawling behaviour (regions highlighted in blue) because we checked that removing a few such trajectories from the subset does not significantly affect our results. Interest- ingly, a few trajectories with high Var(b_t) show signs that the trailing pole could have made contact with the surface, but did not stick for more than a few seconds (example: green highlight). We think it is sensible to call this behaviour walking because the horizontal state is not maintained. We did not investigate this type of event further because of the small sample size and because we have no way to independently verify whether the trailing pole made contact with the surface.

Action: We included the above discussion in revised Online SI (*S9: Aspect ratio profiles*).

Again, we were glad to see this analysis. However, the core of our comment was not the point about ability to segregate tracks in two modes of twitching, but rather that a proper biophysical model of twitching PA motility should be able to recapitulate such transitions. In here, the states are forced by the construction of the model, which limit its predictive abilities (also in the way used in this work, as a model to identify unknown biophysical parameters). See also next point below.

Reply: The bacteria surface interaction during twitching is difficult to measure and not much is reported about it. To avoid making unverifiable assumptions about the interaction, we study walking and crawling separately for the majority of this manuscript. A purely repulsive interaction is used to study the walking state, while a sharp potential well is used to study the crawling state by effectively constraining the z-coordinate of both poles of the crawling bacteria in a plane parallel to the x–y plane. We argue that for a spherocylindrical bacteria on a planar surface, two points of contact provide a simple and reasonable approximation of the un-sticking of the body from the surface. In the section V C. on transitions between walking and crawling we choose a softer surface interaction so that the surface forces become comparable to the TFP mediated forces and the transitions in the simulations are possible.

This reply confirms that some biophysical aspects of motility were forced by hand to correspond to the type of motility observed, with little biological motivation (in the sense of the mechanism that could be responsible). This, on one hand, limits the applicability of the method to other strains and probably even just other conditions for the same strain. On the other hand, it might introduce bias in the model that does not allow the proper discovery/identification of the values of other parameters that can not be measured experimentally. We think it is a clear shortcoming of the method and just need to be acknowledged by the authors as such (if the authors agree of course).

Reply: Based on this comment, we expanded our discussion on the subject of the relative influence of the forces in the system. Our assumption is that viscous forces are much less than friction forces which are less than the maximum forces generated by TFP. Although we are aware of some experiments which use AFM to measure surface interaction forces by measuring the force needed to separate a bacterium from a surface, to our knowledge, the friction forces of *P. aeruginosa* bacteria during twitching have not been adequately measured. Therefore each part of this assumption needs to be argued.

Hierarchy of the forces suggested by the authors in this reply has no experimental evidence. Moreover, it is probably wrong for the simple argument. In this model, there are no other forces than TFP, viscosity and friction. And given that at low Reynolds number regime inertia plays no role, those forces are in balance during motion. That means that friction + viscosity would be just equal to TFP forces. In essence, neglecting those lives no other forces to balance and describe the dynamics between pili attachment/detachment events. And this is unphysical.

Viscous forces: Pönisch et al. (2019) estimate the viscous forces to be approximately 0.2 pN [4] for *N. gonorrhoeae*. Walking *P. aeruginosa* bacteria make out-of-plane rotations which may lead to more significant viscous forces, but it is still reasonable to assume that the system is dominated by TFP forces in most cases. In addition, since crawling is the dominant motility type in this tracking experiment, we are primarily concerned with an accurate simulation of that mode.

This, to our view, is an incorrect statement: what does it mean that motion is dominated by TFP forces? TFP force is the sole active force that generates movement, but in the absence of inertia it needs to be balanced by friction/viscous force! What the authors do by neglecting friction and energy minimization – they disregard realistic dynamics of cell response to pili forces.

Finally, it is true that maximum pili force is of the order of 200pN (as measured in NG bacteria) but the pilus does not have to generate that force if its pulling is only resisted by friction.

Friction forces: Skerker & Berg argue that the interaction between their *P. aeruginosa* and the glass cover-slip is relatively strong, because many of their bacteria did not move despite showing TFP activity. We also see individual bacteria in the tracking data that do not move. One interpretation is that the individual bacteria have a range of surface interaction strengths (which could depend on the expression of adhesive proteins). By focusing on crawling bacteria, we may have selected a subset of the population

with the appropriate surface interaction strength for this motility type. Likewise, the walking bacteria subset may be those with a weaker surface interaction strength. As a parallel argument, in section V.C we demonstrate using our model that a strong surface interaction is necessary for crawling behaviour because of the way TFP pulling forces generate a torque on the cell in this geometry. Although in that case, the force is acting to pull the trailing pole off the surface (un-sticking force), instead of dragging the body across the surface (friction force), we argue these forces should be proportional to some extent. The model TFP have a consistent retraction velocity which we interpret to be the typical retraction velocity under the loads that arise from friction with the surface. The retraction velocity of *P. aeruginosa* TFP has been shown to vary under load by [5] and [6]. Since our approach to modelling is data-driven, and there is no additional data available about the friction force in the experiments we study, we don't make more complex assumptions about how the friction force influences the dynamics. See also our response to comment (5) regarding pili retraction speed.

Slingshots: We do not discuss slingshots as described by Jin et al. (2011) [7] in detail here. This is partly because a computer model has already been reported that reproduces slingshot-like be- haviour [8] and partly because we were not able to reproduce all of the results of Jin et al. (2011) using the data analysis they describe. To be clear, we also see a strong correlation between high velocity displacements and rotations of the cell body in the experimental data we study and in sim- ulations. Simulations show that this behaviour often corresponds to the detachment of a pilus from the surface. When we mention slingshot behaviour in our discussion, we are referring to this corre- lation. Where our data differs from Jin et al. is that we could not find experimental trajectories for which the distribution of velocities of the leading pole is bimodal (in linear or log-scales), forcing us to come up with alternative definitions of slingshot events based on both the velocity and direction of motion. Subsequently, we find that slingshots rarely make up a large fraction of the total contour length of the trajectory for the bacteria in this tracking data.

The fact that slingshots do not take up a significant part of the tracking data is not so important. Inability to describe "proper" slingshots as discussed in Jin 2011 is an indicator of the deficiency of the used model.

Pili retraction velocity: Skerker and Berg measured the retraction velocity of unloaded TFP for the PAK strain of *P. aeruginosa* to be approximately 0.5 μm/s [1] although they note that they ex- pect retraction to be slower under load. This estimate is consistent with 0.64 μm/s measured by Koch et al. (2021) [9] (PA01 strain), and broadly consistent with retraction velocities measured for *N. gonorrhoeae* [10, 11]. Ribbe et al. (2017) measure the retraction velocity of pili for the PA14 strain of *P. aeruginosa* [5]. They measure average retraction velocities of slightly more than 1.1 μm/s under an 8 pN load and note the change in temperature from 29° (Skerker & Berg) to 37° as a reason for the 2× faster retraction rate [5]. Notably, the retraction velocity does fall quite sharply as the load increases in that experiment, suggesting a core difference between *P. aeruginosa* and *N. gonorrhoeae*. Despite the variety of bacteria strains and experimental procedures represented, we originally interpreted these results as a consensus on the average pili retraction velocity of between 0.5 and 1.0 μm/s and concluded that this would be a good estimate for the PA01 *P. aeruginosa* in the data that we study. Although unknown to us at the time of submission, Zhang et al. (2021) measure the retraction velocity of surface attached TFP to be 0.09 μm/s and unbound TFP to be 0.17 μm/s [6] for (PA01) *P. aeruginosa* in very similar experimental conditions to our data. They argue that the bacterial strain (PA01 vs PAK) is the most likely reason for the discrepancy between their results and Skerker & Berg. We studied all of the experimental procedures described in the papers cited here and did not simply take their published results at face value. Our conclusion is that all of these authors most likely give accurate estimates of the pili retraction velocity *in their experiments*, but that this value is actually quite sensitive to a number of environmental factors as well as the specific strain of the bacteria. If we take only measurements on PA01 *P. aeruginosa* bacteria, and only direct observations of TFP that are under load from pulling the cell on the surface, then the most relevant estimate is certainly Zhang et al [6] which is 0.09 μm/s. For reference, our estimate on page 20 of the original draft is 0.132 μm with a 90% confidence interval of [0.074, 0.20] μm/s.

This and the following response, which are quite lengthy, do not address the major and straightforward points we raised. We mentioned that pili retraction velocity is known to depend on load (as authors confirm above) and this is disregarded in the current model. Instead authors argue why it is justified to use lower velocity value.

Pili detachment rate: Tala et al. measure the median surface attachment time of pili of PA01 *P. aeruginosa* to be 1 s (ΔfliC) and 2.3 s (wild-type). Although the unexplained discrepancy be- tween ΔfliC and wild-type is curious, we think these measurements of the pili detachment rate are more relevant to our work than measurements on *N. gonorrhoeae*. Especially considering that it is unclear to what extent the tug-of-war mechanism used to explain the movement of *N. gonorrhoeae* can be applied to *P. aeruginosa*. The tug-of-war mechanism explains how *N. gonorrhoeae* achieves persistent motion despite having TFP distributed all over its body, on the other hand, *P. aeruginosa* achieves persistent motion by a combination of localising its TFP on one pole and sticking its body to the surface to support the crawling state. Tala et al. report surface sensing and catch-bond like mechanisms to explain their observations of TFP surface attachment and retraction in *P. aeruginosa* bacteria, and while we do not think that any single experiment is definitive, we choose to focus on this behaviour in our modelling.

Same here, we wanted to hear the answer on why the force dependent detachment rate of pili is not considered. And no answer to that question is provided above. And there can't be any answer, because the pulling force of the pilus is never even a parameter in the model – see next point.

Reply: Inresponsetothiscomment,weranthesensitivityanalysisagainandincludedtheTFPstall force as a variable parameter. The analysis confirms our intuition that the value of the stall force does not have a strong influence on the simulated trajectories. Due to a lack of good experimental data on the topic, we do not explicitly model the friction forces in the system, instead we assume that friction forces (and viscous forces) in the system are dominated by the TFP mediated forces. As a result, varying the stall force in this model does not lead to qualitatively different motility patterns. When we calibrate the model against experimental data, we predict the TFP retraction speed to be approximately 0.13 $\mu m s^{-1}$. This is our estimate for the retraction speed under the typical loads that arise from dragging the cell across the surface. The fact that this number is supported by experimental measurements is good evidence in support of the model [6].

This is really circular logic to address this point: the pili force has no effect in the model because authors neglect the friction forces and thus (because there is no other forces to balance and relate pili force to) pili force has no effect. Yet in the original biological system, magnitude of the pili force is the central parameter that determines nearly everything: how fast cells move, how frequently pilus detaches, how pili compete to each other if multiple pili bind at the same time!

We believe those points were indeed most important to address and we don't think the authors managed to do so. To conclude, we think that the model used to describe twitching motility essentially neglects nearly everything what is known about pili and TFP motor: a) dependence on the magnitude of the pili force b) force dependent retraction speed c) force dependent detachment rate, and finally there are no other forces than pili and attractive cell-substrate potential and all pili dynamics is reduced to constant speed shortening. As a result, we just can't agree that the prediction of how the pili collectively work to generate the movements is a picture to trust.

Therefore, we can not recommend the publication of the paper in the current form. The authors try to hide the potential oversimplifications of the model by additional lengthy explanations. We think this is misleading. We believe the better strategy would be to admit clearly which known properties of the pili are neglected in the model, and that actually main conclusions on the motility mechanism may be affected by that. The proposed method still can be used as is, but then at least the researchers who decide to use this tool for their tracking data would know what limitations are. And the thing is that those limitations might be good for the considered condition and strain, but what to do in case of other strain, other conditions, or even other bacteria? Develop a new/better model? Probably yes, but this needs to be spelled out in a clear way. That will make the claims of the manuscript less ambitious but more justified.
