# Supplementary material for: Data–driven modelling makes quantitative predictions regarding bacteria surface motility
Source: PLoS Comput Biol. 2024 May 14;20(5):e1012063. doi: 10.1371/journal.pcbi.1012063 (PMC11125545; doi:10.1371/journal.pcbi.1012063)
Supplement: Attachment — Submitted filename: Barton-Reply.pdf [file pcbi.1012063.s014.pdf]

PCOMPBIOL-D-22-01843: **Reply to No. 2**

We thank the referees and the editor for their continuing efforts in reviewing our manuscript. Partially thanks to the persistent critical comments of Reviewer 1 and additional report provided by the Reviewer 3, we realized that some model assumptions have not been sufficiently explained in previous submissions. We hope that we addressed them appropriately in the resubmitted manuscript. We summarize the key comments made by the reviewers, and our actions to address them:

- The first is the introduction of the angular dependent retraction speed of TFP described by the parameter α in our previous submissions. α was originally needed because the speed of TFP retraction that was considered more or less universal in the literature (0.5 to 1 μm/s) was clearly inconsistent with our model and the tracking data we worked with. We introduced it before we became aware of the experiments Zhang et al. measuring the TFP retraction speed while dragging the cell along the surface. Zhang et al. report a lower TFP retraction speed than the previous literature (0.09 μm/s), which does agree with our estimate – without the need to introduce an angular dependence. Based on the responses, we decided it is more natural to use the retraction speed as a free parameter and drop the angular dependence model. We re-run and re-analysed all computations, and updated the results, the figures, and the text accordingly. The results are qualitatively (and in many cases quantitatively) the same, but we have gained a much more relevant prediction: the TFP retraction speed that can be directly compared to the experiments. Since different experiments have been performed with different strains, and at different environmental conditions, we believe that the most relevant experimental comparison for our tracking data are indeed the measurements of Zhang et al., which are consistent with our predictions. We predict different retraction speeds for crawling and walking motility types and which can be naturally explained by a difference in the friction between bacteria body and the surface. Analysis and discussion which uses the angle dependent retraction speed is relegated to supplementary information for reference.

- The second major objection was regarding how we deal with the TFP forces and why friction and viscosity are not directly included in our model. We rewrote the description of the model and added a clear explanation of the model assumptions. In short, we assume that the TFP molecular motor is responsive to the amount of resistance offered by the environment, and adapts the TFP tension in order to achieve a force balance.

  Then, assuming that the motor is sufficiently strong and that the surface is homogeneous, the typical tension in a single pilus that is actively displacing the cell will be fairly constant. We therefore use a constant TFP retraction speed in the simulation as an approximation. However, the retraction speed value is a free parameter, which is optimized to best describe the experimental data.

We hope that these efforts made our manuscript more transparent, convincing, and easier to follow; and that it is now suitable for publication in PLoS Comput. Biol. We highlighted modifications in the revised manuscript (red). The only modification in the supplementary material is the addition of the section (S4) on angular dependence of retraction velocity that we removed from the main text. In the following, we address the comments of the reviewers one by one.

Reviewer 1

We went through the replies of the authors to our previous comments, with realization that not all of them were entirely satisfactory. We comment on them below where appropriate... ... It is clear that various strains and conditions in biological experiments may result in large variability of outcomes. Our point was not to use previously available data, but to produce and analyze the data that allows to validate the approach.

Reply: The request to produce new experimental data seems unreasonable to us, as we are a theoretical and computational group and our focus was on developing and implementing the computational approach for quantitative data–driven modelling of twitching motility. In contrast to the opinion of the referee, we also believe that the available data of Jin et al. provide a good source allowing several non-trivial predictions. In combination with recent measurements of Zhang et al., they form a rare combination where both microscopic TFP-based observations are combined with large scale tracking, which is particularly useful for modelling purposes.

We mentioned Skerker & Berg publication as an example of data that would be particularly important to validate the results of this ms analysis, as they go against previous observations of twitching motility (in whatever bacteria or conditions that has been done). Moreover, those experiment were performed 20 years back and by now with modern imaging techniques both time and space resolution would be significantly higher allowing for proper type of analysis. Therefore, in our opinion, conservative approach would be to admit that the approach still needs validation in what concerns microscopic details of motility, see below... ... While we welcome the additional analysis, the initial assumption that one has to search for straight segments of trajectories is not really justified. If one looks at the movie of the original Skerker & Berg paper – the center of mass of the twitching cell is not traveling on the straight line at all. Actually, we doubt that it would be possible to identify the pili dynamics simply from trajectory characteristics. If only, then by the approach similar to the one advocated in this paper, via proper biophysical model of motility.

Reply: We reiterate that in the data we worked with, which were obtained on a different strain and under different conditions from Berg et al. and others, there are no long displacements – regardless if we search for straight-line or curved-line jumps. It is therefore not a deficiency of our approach if we do not predict them, but rather the property of the observed trajectories. We fully agree with the referee that extracting the TFP dynamics from tracking data is tricky. Again, collecting good quality data from different tracking experiments and performing an analysis of how the twitching mechanism depends on the environmental conditions would be great. The methods developed and used here would be appropriate for the task, however, such meta study is out of scope of the present manuscript.

Again, we were glad to see this analysis. However, the core of our comment was not the point about ability to segregate tracks in two modes of twitching, but rather that a proper biophysical model of twitching PA motility should be able to recapitulate such transitions. In here, the states are forced by the construction of the model, which limit its predictive abilities (also in the way used in this work, as a model to identify unknown biophysical parameters). See also next point below.

This reply confirms that some biophysical aspects of motility were forced by hand to correspond to the type of motility observed, with little biological motivation (in the sense of the mechanism that

could be responsible). This, on one hand, limits the applicability of the method to other strains and probably even just other conditions for the same strain. On the other hand, it might introduce bias in the model that does not allow the proper discovery/identification of the values of other parameters that can not be measured experimentally. We think it is a clear shortcoming of the method and just need to be acknowledged by the authors as such (if the authors agree of course).

Reply: We agree that our model does not describe the transitions from walking to crawling states realistically. To do that, we would need precise information on the adhesion forces between the bacterial body and the surface. To the best of our knowledge, such details are not known, and are extremely difficult to probe experimentally. Therefore, focusing on the experimental trajectories with relatively stable body orientation and comparing them to simulations with very strong (crawling) or very weak (walking) adhesion seems a reasonable way to extract parameters for both distinct types of motion. Varying the surface adhesion strength from very strong to very weak, we have studied the transitions between these two states, but we refer to this part of our results as qualitative, *e.g.*, the trends in the transition rate by changing parameters such as adhesion strength, TFP distribution, or the spawn rate (Fig. 8).

Hierarchy of the forces suggested by the authors in this reply has no experimental evidence. Moreover, it is probably wrong for the simple argument. In this model, there are no other forces than TFP, viscosity and friction. And given that at low Reynolds number regime inertia plays no role, those forces are in balance during motion. That means that friction + viscosity would be just equal to TFP forces. In essence, neglecting those lives no other forces to balance and describe the dynamics between pili attachment/detachment events. And this is unphysical.

Reply: We partially agree with the reviewer's comments in that our description of the model in the previous submission was not perfect. However, we disagree with the claim that our model is unphysical. The reviewer is right that the bacteria TFP pulling forces are balanced by the opposing forces that are due to the dissipation, i.e., friction and viscosity. The assumption in our model is that this is always the case and that the tension in the filaments is adjusted by the action of the molecular motors such that it balances the dissipative forces. It is experimentally established that the TFP retraction speed depends on the tension in the filaments, which is important for the interpretation of our results.

The model description and justification is updated to make it clear that we make minimal assumptions about friction and viscous forces. Simply that these forces affect the retraction speed of TFP, which is model parameter.

This, to our view, is an incorrect statement: what does it mean that motion is dominated by TFP forces? TFP force is the sole active force that generates movement, but in the absence of inertia it needs to be balanced by friction/viscous force! What the authors do by neglecting friction and energy minimization – they disregard realistic dynamics of cell response to pili forces. Finally, it is true that maximum pili force is of the order of 200pN (as measured in NG bacteria) but the pilus does not have to generate that force if its pulling is only resisted by friction

Reply: We agree that the wording "dominated by TFP forces" might have been confusing. Therefore, we removed it from the manuscript.

The fact that slingshots do not take up a significant part of the tracking data is not so important. Inability to describe “proper” slingshots as discussed in Jin 2011 is an indicator of the deficiency of the used model.

Reply: We disagree. Our model is able to describe "proper" slingshots. However, the parameter regime is selected by the tracking data and if there are no (or very few) such events in the data, we cannot and should not expect to see them in the simulations.

This and the following response, which are quite lengthy, do not address the major and straightforward points we raised. We mentioned that pili retraction velocity is known to depend on load (as authors confirm above) and this is disregarded in the current model. Instead authors argue why it is justified to use lower velocity value.

Reply: In the modelling section (section 3) we have clarified the fact that retraction velocity is assumed constant for within a trajectory (see the replies above). We stand by the argument that the lower value of the retraction speed that we predict and was also measured by Zhang et al. (2021) in similar conditions, is likely accurate for the tracking data that we study.

Same here, we wanted to hear the answer on why the force dependent detachment rate of pili is not considered. And no answer to that question is provided above. And there can’t be any answer, because the pulling force of the pilus is never even a parameter in the model – see next point.

Reply: We are aware the the force dependent detachment of TFP is common theme in understanding the twitching mechanics of another species of well studied bacteria, *N. gonorrhoeae*. However, other than the fact that both bacteria use a version of type IV pilus machinery for their motility, we have not yet seen strong evidence that force dependent detachment is a major factor, or that it is essential to understand the twitching of *P. aeruginosa*. More importantly, the surface association time of *P. aeruginosa* is measured by Tala et al. (2019) and Simsek et al. (2023) [1] and neither support a force dependent pilus detachment rate. Naturally, these measurements influence the design of our model, and although it may be interesting to study other cases, we do not think that the data available to us is sufficient to resolve this particular question.

This is really circular logic to address this point: the pili force has no effect in the model because authors neglect the friction forces and thus (because there is no other forces to balance and relate pili force to) pili force has no effect. Yet in the original biological system, magnitude of the pili force is the central parameter that determines nearly everything: how fast cells move, how frequently pilus detaches, how pili compete to each other if multiple pili bind at the same time! We believe those points were indeed most important to address and we don’t think the authors managed to do so. To conclude, we think that the model used to describe twitching motility essentially neglects nearly everything what is known about pili and TFP motor: a) dependence on the magnitude of the pili force b) force dependent retraction speed c) force dependent detachment rate, and finally there are no other forces than pili and attractive cell-substrate potential and all pili dynamics is reduced to constant speed shortening. As a result, we just can’t agree that the prediction of how the pili collectively work to generate the movements is a picture to trust. Therefore, we can not recommend the publication of the paper in the current form. The authors try to hide the potential oversimplifications of the model by additional lengthy explanations. We think this is misleading. We believe the better strategy would be to admit clearly which known properties of the pili are neglected

in the model, and that actually main conclusions on the motility mechanism may be affected by that. The proposed method still can be used as is, but then at least the researchers who decide to use this tool for their tracking data would know what limitations are. And the thing is that those limitations might be good for the considered condition and strain, but what to do in case of other strain, other conditions, or even other bacteria? Develop a new/better model? Probably yes, but this needs to be spelled out in a clear way. That will make the claims of the manuscript less ambitious but more justified.

Reply: We agree that cell-substrate forces and pilus tension would no doubt be very informative in the understanding of twitching behaviour. Unfortunately, no suitable method for measuring these quantities in situ has yet been demonstrated, or provided alongside tracking data. The reason that retraction velocity, and not say, friction force, is used as a parameter in our model is simply because that is a measurable quantity. We hope that the sum of all our replies and actions results in a manuscript where the model assumptions are not "hidden" or "misleading", and that we managed to convince the reviewer that the results obtained have a value and contribute to our understanding of TFP-based surface motility. Regarding whether new or better models could be developed using other, hypothetical data sets, the answer is yes and our code (available online) is appropriate to use as part of such investigations. Besides the new insights into twitching motility, this actually highlights that the computational method we developed is an original result in itself. We believe that our method can be easily applied to both, implementing improved models for twitching motility based on improved experimental data in the future, as well as to other data-driven biological models.

Reviewer 2

The authors have convincingly addressed my concern and adopted their manuscript accordingly. I therefore support publications of the manuscript, once the concerns of the second referee have been addressed.

Reply: We thank the reviewer for positive evaluation and for recommending the manuscript publication

Reviewer 3

J. Dobnikar and coworkers propose a twitching motility model for P. aeruginosa. Using published data, the pilus and cell model is parametrized and a sensitivity analysis is conducted. The authors simulate crawling motility as well as walking motility. The simulation results are compared with experimental data consisting of tracks of migrating bacteria. The strength of this work clearly lies in the computational methodology for finding appropriate model parameters. However, the model itself is very speculative and the predictive value of the model is obviously limited by the many open questions behind the assumptions. In fairness, one can see that the authors did their best to extract as much information from the literature as possible.

Major comments:

A) Clearly, a main weakness of the work is that the model rests on a large number of assumptions

that could not be tested by the authors. Examples highlighting the speculative nature of this model include

1) using the hardly established contact sensing mechanism suggested by Tala et at

Reply: We find the published results of Tala et al. convincing. However, we did consider twitching models that are not dependent on surface sensing. Such models can also describe the experimental trajectories well, but we did not analyse them so extensively. We have added a note in the manuscript in section 3B (page 10) to this effect.

2) assumed van-der-Waals-like surface attachment of the cell

Reply: We use this functional form to generate a potential well that constrains the degrees of freedom in simulations. We are confident that the precise functional form has no quantitative affect.

3) detailed modeling the unknown process of binding between substrate and pili

Reply: We updated the text to mention that pili binding only at their tip is one simplifying assumption and another simplifying assumption is that pili always bind on first contact with the surface.

4) assumed angle-dependent retraction speed

Reply: Based on the experimental evidence by Zhang et al. that for the conditions almost identical to those of our tracking data the TFP retraction speed is substantially lower than previously anticipated, we have eliminated this assumption from our model – as also described in the introduction to our response.

Reply: We repeated all the calculations with a simpler model assuming the TFP retraction speed as a parameter and obtained the optimal retraction speed from parameter inference, which agrees with the experimental observations [2]. We believe that this modification, which is based on the comments in the previous round(s) have made our manuscript more clear and our results more relevant and easy to interpret.

5) assumption that the switching rates measured in Koch et also hold when pili are engaging the substrate

Reply: Although we do rely on Koch et al. measurements to some extent, we also do extensive simulations to estimate the sensitivity of the model to variations in these switching rates.

6) assumed force-independent pilus detachment rate

Reply: We have updated section 3A with a note to the effect that the force-dependence of pilus detachment for *P. aeruginosa* is unclear, and our choice is a simplifying assumption.

7) Assumption that Brownian/active noise does not affect bacterial walking

8) Etc... Some of these assumptions are to the knowledge of the reviewer not appropriate.

Suggestion for A): Please add a comprehensive list of the assumptions, similar to Table 1, where all the assumptions are listed so that a reader can easily understand what the limits of the model are.

Reply: Our approach here is to sure up many places in the text where these assumptions are used, in each case making it clear whether other possibilities could have been considered. In addition we repeated our analysis pipeline to remove the angle-dependent retraction speed entirely, which we feel is justified based on recent and better experimental data. We hope this will have the same or better clarity as a dedicated table because each point can be properly discussed as it appears our model development.

B) The authors emphasize the Bayesian nature of their data analysis. However, the text does not really explain well which techniques are used in practice and the provided GitHub repository does not contain the used routines. This makes it hard to understand what the authors did exactly.

Suggestion for B): since this work is really unique in its effort to use systematic parameter variation, the authors should put more effort into explaining clearly what the analysis routines are doing and provide the code.

Reply: We followed the advise of the reviewer and now provide the scripts in the github repository.

The introduction of the Bayesian method has been slightly expanded to make the motivation more clear. The description of how we evaluate each parameter sample has been re-written.

The approximate Bayesian computation routines are found at https://github.com/dluke/tmos in code/analysis/abcimplement.py. Some additional Jupyter notebooks and input scripts that we used to do this analysis have also been included.

C) There is a very confusing issue regarding stall forces of pili in this manuscript. As previous referees commented, stall forces can play a major role for the pilus dynamics. In Table I, the authors claim that the measures stall forces are 100 pN (Ribbe et al). This is wrong. Ribbe et al measure roughly 30pN. Moreover, while it is shown with sensitivity analysis that the stall forces may not matter much for migration, they may matter for the pilus statistics and retraction speed. The corresponding analysis is lacking.

Suggestion for C): please correct the given stall forces and repeat the simulations with correct value where necessary. A sensitivity analysis regarding the role of stall forces for the pili statistics should be included.

Reply: Thanks for pointing this out, we updated the reference. For these simulations we used an older value for the stall force, commonly reported in the literature, which we believe originates from experiments on *N. gonorrhoeae*. The value of Ribbe et al. is likely the most relevant one, however, this is not crucial for our model and our conclusions. The sensitivity analysis (with stall force between 20 pN and 200 pN) shows that the this parameter has minimal affect on simulated trajectories. Therefore, we are chose not to use additional computational resources to repeat the simulations at a different value of the stall force at this time.

As previous referee points out, the insensitivity of our model to the stall force can be thought of as a consequence of our zero-order approach where we simply provide the retraction speed as a constant

parameter. The model is set up this way because the retraction speed can be measured more readily than the friction forces. We think a more force-centric model would become worthwhile if better force measurements or other relevant data becomes available.

D) The authors emphasize already in the abstract their Mises-Fisher distribution for pili. "...predict previously unresolved distribution of TFP on the bacterial surface and show that these predictions are consistent between the walking and crawling motility..." This "prediction" is does not follow from the authors model but the functional form of the angular pili distribution an assumption. Moreover, the simulations are so complex that it is hard to see how they would confirm the validity of the chosen angular distribution. Thus the statement in the abstract is, in the reviewers opinion, misleading.

Suggestion for D): Please modify statements throughout the manuscript and replace the word "predict" with "suggest a functional form of"

Reply: We agree. The abstract has been re-written to make it clear that we do not estimate the functional form of the TFP distribution but its width (assuming a generic Gaussian-like form).

E) Pole switching: It is well-known that Pseudomonads regularly switch their pole, which could affect the migration dynamics (e.g. Oliveira et al PNAS 113.23 (2016): 6532-6537.)

Reply: We carefully analysed our tracking data for evidence of this behaviour and found none. Perhaps the experimental conditions were not amenable to pole switching.

Suggestion for E): The authors may repeat their simulations by including reversal. How does this effect change the simulation results regarding MSDs in the authors' work?

F) Tracking: Tracked trajectories are very variable and the authors note correctly that tracking of walking cells is challenging. One cannot avoid the sense that the authors have not invested enough care in generating the correct statistics since (i) "trajectories are selected by hand", which amount to uncontrolled removal of outliers, (ii) no errorbars are given, (ii) only a few trajectories are used.

Suggestion for F): Ideally, the tracking should be repeated for more data without manual removal of trajectories, in particular for the walking cells. At the very minimum, the analysis should be redone without manual removal of trajectories and those results should be compared to the previous results where trajectories were removed manually. Statistics and errorbars should be provided.

Reply: We originally developed both automated and manual approaches for filtering trajectories, but described only the latter. In the interest of reproducibility, we have repeated our analysis with the former. The simple procedure used for filtering experimental trajectories from the data is described in section 4. We repeated our entire analysis so that it uses this automatic procedure.

G) A recent biorXiv preprint by Simsek et al. https://doi.org/10.1101/2023.05.09.538458 clarifies through experimental data and modeling most of the questions that arise in the context of this model (including pili angles, rate parameters, adhesion, etc). The authors are encouraged to discuss their model in the light of these new findings.

Reply: Thanks to the reviewer for making us aware of this recent work which supports several of our conclusions. We now cite it in our discussion on the apparent ratio of TFP that contribute to

twitching and also when we discuss the apparently essential role of passive surface adhesion. We also cite this paper in support of some of the assumptions that we make when introducing our model.

Minor comments: - P. Aeruginosa -> P. aeruginosa in main text and SI

- In the SI, the authors state that they use uniform priors for their "Bayesian" analysis. However, since uniform priors are used, this is nothing but a standard parameter variation. Please clarify this in the main text and explain which aspects of Bayesian analysis are used.

Reply: Our use of the term Bayesian is consistent with other works using uniform priors, e.g. [3].

-The persistence length of pili is a matter of dispute and very different quantities have been reported. While the author's choice to use the value of 5micrometer is appealing, it would be appropriate to cite some earlier work on this topic, e.g., de Haan HW. Biophys J. 2016 Nov 15;111(10):2263-2273 or Lu, Shun, et al. Biophys. J 108.12 (2015): 2865-2875.

Reply: We cite these papers in the modelling and sensitivity analysis sections where appropriate. We added a line to the text to make it clear that we do not investigate the full range of persistence length values that have been reported in the literature at one time or another.

References

[1] A. N. Simsek, M. D. Koch, J. Sanfilippo, Z. Gitai, G. Gompper, and B. Sabass, "Type-iv pili tune an adhesion-migration trade-off during surface colonization of pseudomonas aeruginosa," *bioRxiv*, pp. 2023–05, 2023.

[2] J. Zhang, S. Li, T. Sun, Y. Zong, W. Zhang, and K. Zhao, "A simple, switchable pili-labelling method by plasmid-based replacement of pilin," *Environmental Microbiology*, vol. 23, no. 5, pp. 2692–2703, 2021.

[3] J. Kursawe, R. E. Baker, and A. G. Fletcher, "Approximate bayesian computation reveals the importance of repeated measurements for parameterising cell-based models of growing tissues," *Journal of theoretical biology*, vol. 443, pp. 66–81, 2018.
