# Supplementary material for: Data–driven modelling makes quantitative predictions regarding bacteria surface motility
Source: PLoS Comput Biol. 2024 May 14;20(5):e1012063. doi: 10.1371/journal.pcbi.1012063 (PMC11125545; doi:10.1371/journal.pcbi.1012063)
Supplement: Attachment — Submitted filename: Barton_Response_3.pdf [file pcbi.1012063.s015.pdf]

PCOMPBIOL-D-22-01843: **Reply to the Referees No. 3**

Daniel Barton, Yow-Ren Chang, William Ducker, Jure Dobnikar

March 27, 2024

Dear Editor,

thank you for another round of the review comments. We are thankful to the reviewers for their persistent interest in making our manuscript more readable and precise. We have addressed their latest comments and we hope that the manuscript is now suitable for publication. Below, we address the comments one-by-one.

Sincerely, the authors.

Reviewer #1: I reread the manuscript from the scratch. In order not to go into another round of back and forth with the authors, I think I can state that the current version of the manuscript provides clearly the details of how simulations are done and how the model is used to infer the twitching motility characteristics. I still believe that the model is not critically discussed with respect to the assumptions made. Instead we mostly presented with how good it matches the experimental data. I can't understand why the authors are so reluctant to do that critical assessment, given that it was never a barrier for this work to be published. For example, a suggestion of the Reviewer 3 with the table of assumptions would work, or a subsection doing the same. Being critical and discussing limitations is what stimulates further research and leads towards novel discoveries. All that being said, I also think that a somewhat informed reader would be able to recognize the limitations of the model by carefully reading this manuscript, and would be able to judge the reliability of the results or to adjust/modify the method for their own use/data. Therefore I don't want to use our arguments from previous rounds of review to stop this work from being published and I leave it up to editors if a concise critical discussion of the model limitations need to be added to the manuscript.

Reply: We thank the reviewer for another critical evaluation of our manuscript and for the conclusion that the manuscript is in principle suitable for publication. Since both reviewers insist on the changes regarding the discussion of the model assumptions, we have decided to follow the advice of the reviewer 3 and added a table summarizing and shortly discussing our assumptions. We also added a couple of additional discussions that are marked in the resubmitted manuscript. We hope that this addresses the final concerns of the reviewer.

Reviewer #3: The authors have made some effort to respond to the comments from the reviewer. However, some aspects remain unsatisfactory and really should be fixed so that the otherwise interesting work can be better appreciated by the community.

1. The authors now use the very low value of retraction speed for anchored pili measured in Zhang et al. Readers may buy that this is appropriate for the experiments used as reference for this work. However, the experiments are almost not explained in the main text. Do the authors use the data from Ni et al.? Regardless where the data comes form, the experiments need to be explained in detail since only that will allow the reader to understand the assumptions. At which temperature were the experiments conducted that the authors analyzed? What was the setup? The microscope? Which medium was used? Were the experiments conducted in Log-phase? etc etc etc

Reply: We have followed the advise of the reviewer and added a paragraph explaining the conditions under which the experiments were performed. This hopefully clearly demonstrates that the experiments of Zhang et al., where they measure the lower retraction speed for anchored TFP, are indeed the only such measurement performed at almost identical conditions to the tracking data of Ni et al., which we use in our analysis.

2. The authors seem to have a lot of confidence in the relevance of a "tip-sensing mechanism" suggested by Tala et al. Although they admit that it seems hard to prove tip sensing, the authors now add a cryptic sentence claiming that tip sensing is necessary in their simulations. Without substantial evidence, including data and simulation analysis, such a statement makes no sense in the Reviewer's opition. Please either provide that evidence or remove the statement.

Please note that Tala et al. could only analyze extremely long pili due to imaging artifacts caused by the cell body. The delay times measured for the "rare-event long pili" are hardly representative of what's really going on. Tip sensing has neither been reported in extensive previous work with optical traps, nor in later work with labeled pili. Thus, it must be seen as highly speculative.

Reply: We see that the following sentence from section 3.B in our previous submission was indeed confusing: "*...In this work, we assume that the reported surface sensing mechanism exists, for further discussion see S6 Appendix...*", since in the appendix 6, we argue that models without the tip-sensing mechanism also produce trajectories similar to the experimental data. Models without tip-sensing usually have a larger average number of TFP, with the additional TFP being in passive or extension states and therefore not contributing much to the active movement of the cell. In other words, our analysis does not provide evidence for, or against, the surface sensing mechanism, and since the sensitivity analysis shows that the parameter (delay time) regulating this mechanism is not a sensitive quantity, we chose to use a model with a short delay time, *i.e.,* with surface-sensing. We have removed the confusing sentence and added a short paragraph explaining these arguments better.

3. The author's didn't bother to respond to the question whether Brownian noise affects the motion of cells. While it is clear that tightly adherent cells are not affect by this noise, walking bacteria may transiently detach from the surface. Such jumps may strongly affect the nature of the diffusive random walk. Please analyze the data regarding this point and provide a conclusive statement on whether this is relevant for your experiments.

Reply: We thank the referee for insisting on this point, we indeed fail to reply to this question in the previous resubmission. In our analysis, we assumed that the Brownian motion does not present a large contribution to the displacements of bacteria at the surface. As the referee points out, for cells that are strongly adhered to the surface, this is a reasonable assumption and therefore it is

straightforward to apply it to the crawling trajectory subset (which is the main focus of our analysis). If bacteria also adhere to the surface most of the time during walking, than at least their leading pole is not affected by the Brownian noise, while the trailing pole can undergo rotational diffusion, and potentially different velocities could be observed for both poles, but the analysis of the leading pole motility without Brownian component is appropriate.

If during walking the cells completely unbind from the surface, they would diffuse due to Brownian motion. Estimates for a diffusion constant of a 3 micron elongated object in aqueous solution close to a hard surface are in the range of $10^{-3} - 10^{-2} \mu\mathrm{m}^2/\mathrm{s}$ and a typical Brownian displacement in a given time interval Δt is $\langle r \rangle = \sqrt{2D\Delta t}$ (for 2D diffusion). In our analysis of the tracking data, we discretize the trajectories with a fixed step length of 0.12 $\mu\mathrm{m}$, and observe a median time for the walking and crawling trajectories to be 0.5 s and 1.6 s, respectively. An expected Brownian displacement corresponding to a typical walking median time interval of 0.5 s is below 0.1 $\mu\mathrm{m}$, *i.e.*, about 2 to 4 times smaller than the length scale 0.12 $\mu\mathrm{m}$ in our data. Therefore, we expect that the thermal noise contributions – if they exist – contribute to lower speed events. The difference, however, is not sufficiently large to dismiss the Brownian diffusion as a source for observed displacements in our analysis, and in principle it is non-trivial to separate pili-driven and potential thermal fluctuations-driven displacements during walking.

In the absence of pili-resolved imaging during the tracking experiments, it is difficult to directly detect potential detachments of the cell from the surface. However, we anticipate that if the cells were to completely detach, they would be free to rotate and to move away from the surface. This would result in them getting out of focus etc. We do not observe such events in the datasets we use, which suggests that the cells are mostly adhered to the surface (this could be either by TFP or by other membrane molecules). Moreover, our model predicts that on average more TFP are bound to the surface in the walking (around 5 on average) than in the crawling (around 2 on average) mode. If this prediction is realistic, it is highly unlikely to expect all TFP to be unbound at a given time (since this argument is built on the assumption, it is more a consistency check than a justification of the assumption).

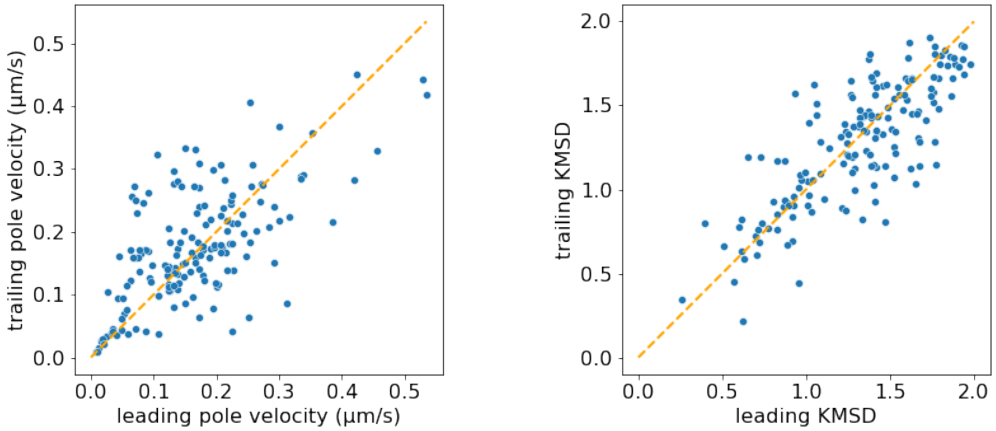

Finally, following the comments of the referee, we have performed some further analysis of the

tracking data for walking trajectories. We examined the statistics of displacements of the trailing and the leading pole in the walking mode, and found no significant difference between the distribution of the speed, nor between the mean square displacements (MSD) for both poles. However, the median scaling exponent of the MSD is 1.3 to 1.4, which is a clear evidence that the diffusion driven by the thermal noise is not a dominant mechanism for observed walking motility.

Having said that, not all the arguments above are bulletproof, and the detached configurations might be present in the trajectories at least for short time intervals. This potentially renders our observations of the walking behaviour less precise than that of the crawling motility. This, combined with the fact that the walking trajectories are inherently less uniform and more noisy, was the main reason why we focused more on the crawling behaviour in the present manuscript.

4. Since Reviewer 1 seems to share my concerns regarding the intransparency of the model, I would like to repeat my suggestion regarding a comprehensive table summarizing the model assumptions to the main text. I really feel that this table would improve the transparency of the work.

Reply: We finally followed the advise and added the table of model assumptions, even though we believe that the assumptions had been sufficiently described in the text already. Although not a usual practise, we do agree that in our case of a complex model with many assumptions and parameters, such a table improves the readability of the manuscript.
